# Supplementary material for: A systematic review and meta-analysis of the potential non-human animal reservoirs and arthropod vectors of the Mayaro virus
Source: PLoS Negl Trop Dis. 2021 Dec 13;15(12):e0010016. doi: 10.1371/journal.pntd.0010016 (PMC8699665; doi:10.1371/journal.pntd.0010016)
Supplement: S1 Table — Includes all positive samples regardless of test method. (DOCX) [file pntd.0010016.s002.docx]

**S1 Table.** **MAYV positivity by taxa of wild mammals and reptiles in included studies**

| **Family** | **Genus** | **Species** | **Common Name** | **MAYV Positive Samples** | **Total Positive** | **Total Tested^#^** | **Country of study*** |
| --- | --- | --- | --- | --- | --- | --- | --- |
| ***Order: Primate*** | | | | | | | |
| Aotidae | *Aotus* | *A. trivirgatus* | Three-striped night monkey | No | 0 | 6 | Panama [1, 2] |
|  |  | NA | NA | Yes | 1 | 4 | Colombia **[3]*** |
| Atelidae | *Alouatta* | *A. belzebul* | Red-handed howler | Yes | 1 | 1 | Brazil **[4]*** |
|  |  | *A. caraya* | Black Howler | Yes | 0 | 97 | Brazil [5, 6], Argentina/Paraguay [7] |
|  |  | *A. seniculus* | Venezuelan red howler | Yes | 52 | 99 | Peru **[8]***,  French Guiana **[9]*** |
|  |  | *A. villosa* | Guatemalan black howler | Yes | 3 | 5 | Panama **[1]*** [2] |
|  |  | NA | NA | Yes | 7 | 11 | Colombia **[3]*** |
|  | *Ateles* | *A. marginatus* | White-cheeked spider monkey | Yes | 1 | 1 | Brazil **[10]*** |
|  |  | NA | NA | No | 0 | 5 | Colombia [3] |
|  | *Lagothrix* | *L. poeppigii* | Silvery wooly monkey | Yes | 6 | 11 | Peru **[8]*** |
| Callithricidae | *Callithrix* | *C. jacchus* | Common marmoset | No | 0 | 1 | Brazil [10] |
|  |  | *C. argentata* | Silvery marmoset | Yes | 32 | 119 | Brazil **[4]*** |
|  |  | *C. penicillata* | Black-tufted marmoset | No | 0 | 3 | Brazil [6] |
|  | *Leontopithecus* | *L. chrysomelas* | Golden-headed lion tamarin | No | 0 | 103 | Brazil [11] |
|  | *Saguinas* | *S. oedipus* | Cotton-top tamarin | No | 0 | NA | Panama [2] |
|  |  | *S. geoffroyi* | Geoffrey’s tamarin | No | 0 | 32 | Panama [1] |
|  |  | *S. midas* | Red-handed tamarin | Yes | 8 | 42 | French Guiana **[9]*** |
| Cebidae | *Cebus* | *C. apella* | Tufted capuchin | Yes | 10 | 62 | Brazil **[5]*** |
|  |  | *C. capucinus* | Colombian white-faced capuchin | No | 0 | 1 | Panama [1, 2] |
|  |  | *C. albifrons* | White-fronted capuchin | No | 0 | 2 | Peru [8] |
|  |  | *C. libidinosus* | Black-striped capuchin | Yes | 6 | 142 | Brazil **[12]*** [6] |
|  |  | NA | NA | Yes | 4 | 13 | Colombia **[3]*** |
|  | *Sapajus* | *S. macrocephalus* | Large-headed capuchin | Yes | 1 | 6 | Peru **[8]*** |
|  |  | *S. flavius* | Blond capuchin | No | 0 | 32 | Brazil [10, 12] |
|  |  | *S. robustus* | Crested capuchin | No | 0 | 1 | Brazil [10] |
|  |  | *S. xanthosternos* | Golden-bellied capuchin | Yes | 1 | 2 | Brazil **[10]***[11] |
|  |  | NA | NA | Yes | 3 | 48 | Brazil **[13]*** [10] |
|  | *Saimiri* | *S. macrodon* | Ecuadorian squirrel monkey | No | 0 | 3 | Peru [8] |
|  |  | *S. sciureus* | Common squirrel monkey | Yes | 4 | 6 | French Guiana **[9]*** |
|  |  | NA | NA | Yes | 1 | 1 | Colombia **[3]*** |
| Pitheciidae | *Cacajao* | *C. calvus* | Bald uakari | Yes | 1 | 3 | Peru **[8]*** |
|  | *Callicebus* | *C. brunneus* | Brown titi | Yes | 1 | NA | Brazil **[14]*** |
|  |  | *C. donacophilus* | White-eared titi | No | 0 | 1 | Brazil [5] |
|  | *Pithecia* | *P. monachus* | Monk saki | No | 0 | 1 | Peru [8] |
|  |  | *P. pithecia* | White-faced saki | Yes | 4 | 5 | French Guiana **[9]*** |
| NA | NA | NA | NA | Yes | 7 | 53 | Brazil **[15]*[14]*[16]***, Panama/Colombia [17] |
| ***Order: Rodentia*** | | | | | | | |
| Cricetidae | *Neacomys* | *N. guianae* | Guianan neacomys | No | 0 | NA | Brazil [18] |
|  | *Nectomis* | *N. squamipes* | Atlantic Forest nectomys | No | 0 | NA | Brazil [18] |
|  | *Oryzomys* | *O. alfaroi* | Alfaro’s rice rat | No | 0 | 9 | Colombia [19] |
|  |  | *O. caliginosus* | Costa Rican dusky rice rat | No | 0 | 47 | Colombia [19] |
|  |  | *O. goeldi* | Large-headed rice rat | No | 0 | NA | Brazil [18] |
|  |  | *N/A* | NA | No | 0 | 458 | Panama/Colombia [17] |
|  | *Sigmodon* | *S. hispidus* | Hispid cotton rat | No | 0 | 183 | Panama [1, 20] |
|  | *Oxymycterus* | *O. amazonicus* | Amazonian hocicudo | No | 0 | NA | Brazil [18] |
|  | *Rhipidomys* | *R. latimanus* | Broad-footed climbing mouse | No | 0 | 7 | Colombia [19] |
|  | *Zygodontomys* | *Z. brevicauda* | Common cane mouse | No | 0 | 1 | Colombia [19] |
|  | NA | NA | NA | No | 0 | 150 | Brazil [4] |
| Cuniculidae | *Agouti* | *A. paca* | Lowland paca | Yes | 1 | 29 | Peru **[8]*,**  French Guiana [9]**,** Panama [1, 2], |
| Dasyproctidae | *Dasyprocta* | *D. fuliginosa* | Black agouti | Yes | 3 | 27 | Peru **[8]*** |
|  |  | *D. leporina* | Red-rumped agouti | Yes | 5 | 29 | French Guiana **[9]*** |
|  |  | *D. punctata* | Central American agouti | Yes | 3 | 5 | Panama **[1]*** [2] |
|  | *Myoprocta* | *M. acouchy* | Red acouchi | No | 0 | 29 | French Guiana [9] |
| Echimyidae | *Echimys* | NA | NA | Yes | 1 | 21 | French Guiana **[9]*** |
|  | *Proechimys* | *P. guyannensis* | Guyenne spiny rat | No | 0 | 2 | Brazil [18, 21] |
|  |  | *P. longicaudatus* | Long-tailed spiny rat | No | 0 | NA | Brazil [18] |
|  |  | *P. semispinosus* | Tome's spiny rat | No | 0 | 76 | Panama [1, 2, 20] |
|  |  | NA | NA | Yes | 1 | 149 | French Guiana **[9]*,**  Peru [8], Panama/Colombia [17], |
|  | NA | NA | N/A | No | 0 | 85 | Brazil [4] |
| Erethizontidae | *Coendou* | *C. melanurus* | Black-tailed hairy dwarf porcupine | Yes | 2 | 15 | French Guiana **[9]*** |
|  |  | *C. prehensilis* | Brazilian porcupine | Yes | 3 | 26 | French Guiana **[9]*** |
|  |  | *C. rothschildii* | Rothschild’s porcupine | No | 0 | 1 | Panama [1, 2] |
| Sciuridae | *Sciurus* | *S. granatensis* | Red-tailed squirrel | No | 0 | 11 | Panama [1, 2] |
|  |  | *S. igniventris* | Northern Amazon red squirrel | No | 0 | 1 | Peru [8] |
|  | NA | NA | NA | No | 0 | 2 | Brazil [4] |
| Muridae | *Mus* | *M. musculus* | House mouse | No | 0 | 2 | Colombia [19] |
|  | *Rattus* | *R. norvegicus* | Brown rat | No | 0 | 2 | Colombia [19] |
|  |  | *R. rattus* | Black rat | No | 0 | 40 | Brazil [18],  Colombia [19] |
|  |  | NA | NA | No | 0 | 2 | Colombia [19] |
| NA | NA | NA | NA | Yes | 71 | 1092 | Brazil **[15]*** [16] Panama/Colombia [17] |
| ***Order: Chiroptera*** | | | | | | | |
| Molossidae | *Molossus* | *M. ater* | Black mastiff bat | No | 0 | 165 | Trinidad [22] |
|  |  | *M. molossus* | Velvety free-tailed bat | No | 0 | 41 | Trinidad [22] |
|  | NA | NA | NA | No | 0 | 2 | Brazil [4] |
| Mormoopidae | *Pteronotus* | *P. davyi* | Davy’s naked-backed bat | No | 0 | 18 | Trinidad [22] |
|  |  | *P. parnellii* | Parnell’s mustached bat | No | 0 | 30 | Trinidad [22] |
| Natalidae | *Natalus* | *N. tumidirostris* | Trinidadian funnel-eared bat | No | 0 | 10 | Trinidad [22] |
| Phyllostomidae | *Anoura* | *A. geoffroyi* | Geoffroy’s tailless bat | No | 0 | 31 | Trinidad [22] |
|  | *Artibeus* | *A. cinereus* | Gervais’s fruit-eating bat | No | 0 | 22 | Trinidad [22] |
|  |  | *A. jamaicensis* | Jamaican fruit bat | No | 0 | 93 | Trinidad [22],  Panama [1] |
|  |  | *A. lituratus* | Great fruit-eating bat | No | 0 | 66 | Trinidad [22],  Panama [1],  Brazil [18],  Colombia [19] |
|  |  | *A. hartii* | Velvety fruit-eating bat | No | 0 | 1 | Colombia [19] |
|  | *Carollia* | *C. perspicillata* | Seba’s short-tailed bat | No | 0 | 192 | Trinidad [22],  Panama [1],  Brazil [18],  Colombia [19] |
|  | *Chiroderma* | *C. villosum* | Hairy big-eyed bat | No | 0 | 1 | Colombia [19] |
|  | *Glossophaga* | *G. soricina* | Pallas’s long-tongued bat | No | 0 | 47 | Trinidad [22],  Brazil [18],  Colombia [19] |
|  | *Phyllostomus* | *P. hastatus* | Greater spear-nosed bat | No | 0 | 170 | Trinidad [22] |
|  |  | *P. discolor* | Pale spear-nosed bat | No | 0 | N/A | Brazil [18] |
|  | *Sturnira* | NA | NA | No | 0 | 21 | Trinidad [22] |
|  |  | *S. lilium* | Little yellow-shouldered bat | No | 0 | 2 | Colombia [19] |
|  | *Vampyrops* | *V. helleri* | Heller’s broad-nosed bat | No | 0 | 30 | Trinidad [22] |
|  | NA | NA | NA | No | 0 | 172 | Brazil [4] |
| NA | NA | NA | NA | No | 0 | 431 | Panama [1], Panama/Colombia [17], Brazil [16] |
| ***Order: Cingulata*** | | | | | | | |
| Chlamyphoridae | *Cabassous* | *C. centralis* | Northern naked-tailed armadillo | No | 0 | 1 | Panama [1, 2] |
| Dasypodidae | *Dasypus* | *D. kappleri* | Greater long-nosed armadillo | No | 0 | 20 | French Guiana [9] |
|  |  | *D. novemcinctus* | Nine-banded armadillo | Yes | 6 | 48 | Peru **[8]***,  French Guiana **[9]***, Panama [1, 2] |
|  | NA | NA | NA | No | 0 | 1 | Brazil [4] |
| ***Order: Didelphimorphia*** | | | | | | | |
| Didelphidae | *Caluromys* | *C. philander* | Bare-tailed woolly opossum | Yes | 1 | 5 | French Guiana **[9]***, Brazil [18] |
|  |  | *C. derbianus* | Derby's wooly opossum | No | 0 | 3 | Colombia [19] |
|  | *Didelphis* | *D. albiventris* | White-eared opossum | Yes | 2 | 19 | French Guiana **[9]***, Brazil [18] |
|  |  | *D. marsupialis* | Common opossum | Yes | 1 | 87 | French Guiana **[9]***, Panama [1, 2],  Brazil [18, 21],  Colombia [19] |
|  |  | NA | NA | No | 0 | 43 | Panama/Colombia [17] |
|  | *Marmosa* | *M. cinerea* | Woolly mouse opossum | No | 0 | NA | Brazil |
|  |  | *M. murina* | Linnaeus's mouse opossum | No | 0 | 2 | Colombia [19] |
|  |  | NA | NA | Yes | 7 | 296 | Brazil **[15]***  Panama [1], Panama/Colombia [17], |
|  | *Metachirus* | *M. nudicaudatus* | Brown four-eyed opossum | No | 0 | 21 | Panama [1],  French Guiana [9],  Brazil [18] |
|  |  | NA | NA | No | 0 | 37 | Panama/Colombia [17] |
|  | *Monodelphis* | *M. americana* | Northern three-striped opossum | No | 0 | NA | Brazil [18] |
|  | *Philander* | *P. opossum* | Gray four-eyed opossum | Yes | 5 | 27 | French Guiana **[9]***, Brazil [18] |
|  |  | *P. nudicaudatus* | Gray and black four-eyed opossum | No | 0 | NA | Panama [2] |
|  | NA | NA | NA | Yes | 9 | 303 | Brazil **[15]*** [4]**,** Panama/Colombia [17] |
| ***Order: Carnivora*** | | | | | | | |
| Canidae | *Dusicyon* | *D. thous* | Crab-eating fox | No | 0 | 1 | Colombia [19] |
| Mustelidae | *Eira* | *E. barbara* | Tayra | No | 0 | N/A | Panama [2] |
|  | NA | NA | NA | No | 0 | 3 | Brazil [4] |
| Procyonidae | *Bassaricyon* | *B. gabii* | Olingo | No | 0 | NA | Panama [2] |
|  | *Bassariscus* | *B. sumichrasti* | Cacomistle | No | 0 | NA | Panama [2] |
|  | *Nasua* | *N. nasua* | South American coati | No | 0 | 9 | Panama [1, 2],  Peru [8] |
|  | *Potos* | *P. flavus* | Kinkajou | Yes | 1 | 16 | French Guiana **[9]***, Panama [1, 2] |
|  | *Procyon* | *P. canerivorus* | Crab-eating raccoon | No | 0 | NA | Panama [2] |
|  | NA | NA | NA | No | 0 | 4 | Brazil [4] |
| ***Order Artiodactyla*** | | | | | | | |
| Cervidae | *Mazama* | *M. americana* | Red brocket | No | 0 | 3 | Peru [8],  Panama [2] |
|  |  | NA | NA | No | 0 | 10 | French Guiana [9] |
| Tayassuidae | *Pecari* | *P. tajacu* | Collared peccary | Yes | 1 | 13 | Peru **[8]***,  French Guiana [9], Panama [2] |
| ***Order Crocodilia*** | | | | | | | |
| Alligatoridae | NA | NA | Caiman | No | 0 | 87 | Brazil [23] |
| ***Order Lagomorpha*** | | | | | | | |
| Leporidae | *Sylvilagus* | *S. brasiliensis* | Tapeti | No | 0 | 15 | Panama [1, 2] |
|  | *N/A* | *N/A* | N/A | No | 0 | 24 | Panama/Colombia [17] |
| ***Order Pilosa*** | | | | | | | |
| Bradypodidae | *Bradypus* | *B. variegatus* | Brown-throated sloth | No | 0 | 69 | Costa Rica [24],  Panama [1, 2],  Brazil [11] |
|  |  | *B. tridactylus* | Pale-throated sloth | Yes | 1 | 29 | French Guiana **[9]***, Brazil [18] |
|  |  | *B. torquatus* | Maned sloth | No | 0 | 22 | Brazil [11] |
|  |  | *N/A* | N/A | Yes | 1 | 4 | Brazil **[4]*,**  Peru [8] |
|  | *N/A* | *N/A* | N/A | No | 0 | 11 | Brazil [4] |
| Choloepodidae | *Choloepus* | *C. hoffmanni* | Hoffmann's two-toed sloth | No | 0 | 96 | Costa Rica [24],  Panama [1, 2] |
|  |  | *C. didactylus* | Linnaeus's two-toed sloth | Yes | 7 | 26 | French Guiana **[9]***, Brazil [18] |
| Myrmecophagidae | *Tamandua* | *T. mexicana* | Northern tamandua | No | 0 | NA | Panama [2] |
|  |  | *T. tetradactyla* | Southern tamandua | Yes | 6 | 40 | French Guiana **[9]***, Panama [1] |
| ***Order Squamata*** | | | | | | | |
| Teiidae | *Ameiva* | *A. ameiva* | South American ground lizard | Yes | 1 | NA | Brazil **[4]*** |
| Tropiduridae | *Tropidurus* | *T. torquatus* | Amazon lava lizard | Yes | 1 | NA | Brazil **[4]*** |
| Iguanidae | *Iguana* | NA | Iguana | No | 0 | 1 | Colombia [19] |
| NA | NA | NA | Lizard | No | 0 | 1 | Colombia [19] |
| ***Order Testudines*** | | | | | | | |
| Testudinidae | *Geochelone* | *G. denticulata* | Yellow-footed tortoise | No | 0 | NA | Brazil [18] |

MAYV: Mayaro virus

^#^Total is pooled across all studies. A value of NA indicates that a study reported testing an animal for MAYV but did not specify how many were tested.

^*^Indicates the location where the positive animal was found and the citation for the study that reported the positive animal.

References

1. Seymour C, Peralta PH, Montgomery GG. Serologic evidence of natural togavirus infections in Panamanian sloths and other vertebrates. Am J Trop Med Hyg. 1983;32(4):854-61. Epub 1983/07/01. doi: 10.4269/ajtmh.1983.32.854. PubMed PMID: 6309027.

2. Galindo P, Adames A, Peralta P, Johnson C, Read R. Impacto de la hidroeléctrica de Bayano en la transmisión de arbovirus. Rev Med Pan. 1983;8:89-134.

3. Groot H. Estudios sobre virus transmitidos por artropodos en Colombia. Rev Acad Colomb Cienc. 1964;12(46):191-217. doi: 10.18257/raccefyn.565.

4. Hoch AL, Peterson NE, LeDuc JW, Pinheiro FP. An outbreak of Mayaro virus disease in Belterra, Brazil. III. Entomological and ecological studies. Am J Trop Med Hyg. 1981;30(3):689-98. Epub 1981/05/01. doi: 10.4269/ajtmh.1981.30.689. PubMed PMID: 6266265.

5. Batista PM, Andreotti R, Chiang JO, Ferreira MS, Vasconcelos PF. Seroepidemiological monitoring in sentinel animals and vectors as part of arbovirus surveillance in the state of Mato Grosso do Sul, Brazil. Rev Soc Bras Med Trop. 2012;45(2):168-73. Epub 2012/04/27. doi: 10.1590/s0037-86822012000200006. PubMed PMID: 22534986.

6. Gibrail MM. Detecção de anticorpos para arbovirus em primatas não humanos no município de Goiânia, Goiás [M.Sc. Thesis]. Goiânia: Universidade Federal de Goiás; 2015. Available from: <https://repositorio.bc.ufg.br/tede/handle/tede/5552>.

7. Diaz LA, Diaz Mdel P, Almiron WR, Contigiani MS. Infection by UNA virus (Alphavirus; Togaviridae) and risk factor analysis in black howler monkeys (Alouatta caraya) from Paraguay and Argentina. Trans R Soc Trop Med Hyg. 2007;101(10):1039-41. Epub 2007/07/31. doi: 10.1016/j.trstmh.2007.04.009. PubMed PMID: 17658571.

8. Perez JG, Carrera JP, Serrano E, Pitti Y, Maguina JL, Mentaberre G, et al. Serologic Evidence of Zoonotic Alphaviruses in Humans from an Indigenous Community in the Peruvian Amazon. Am J Trop Med Hyg. 2019. Epub 2019/10/02. doi: 10.4269/ajtmh.18-0850. PubMed PMID: 31571566.

9. de Thoisy B, Gardon J, Salas RA, Morvan J, Kazanji M. Mayaro virus in wild mammals, French Guiana. Emerg Infect Dis. 2003;9(10):1326-9. Epub 2003/11/12. doi: 10.3201/eid0910.030161. PubMed PMID: 14609474; PubMed Central PMCID: PMCPMC3033094.

10. Moreira-Soto A, Carneiro ID, Fischer C, Feldmann M, Kummerer BM, Silva NS, et al. Limited Evidence for Infection of Urban and Peri-urban Nonhuman Primates with Zika and Chikungunya Viruses in Brazil. mSphere. 2018;3(1). doi: 10.1128/mSphere.00523-17. PubMed PMID: WOS:000425277500024.

11. Catenacci LS. Abordagem one health para vigilância de arbovirus na Mata Atlântica do sul da Bahia, Brasil. [Ph.D. Thesis]. Ananindeua: Instituto Evandro Chagas; 2017. Available from: <https://patua.iec.gov.br/handle/iec/3073>.

12. Laroque PO, Valença-Montenegro MM, Ferreira DRA, Chiang JO, Cordeiro MT, Vasconcelos PFC, et al. Levantamento soroepidemiológico para arbovírus em macaco-prego-galego (Cebus flavius) de vida livre no estado da Paraíba e em macaco-prego (Cebus libidinosus) de cativeiro do nordeste do Brasil. Pesq Vet Bras. 2014;34:462-8.

13. Paulo M, Renato A, Da Carneiro Rocha T, Eliane C, Navarro da Silva M. Serosurvey of arbovirus in free-living non-human primates (Sapajus spp.) in Brazil. J Environ Anal Chem. 2015;2(155):2380-91.1000155.

14. Degallier N, Travassos da Rosa AP, Vasconcelos PFC, Hervé JP, Sa Filho GC, Travassos da Rosa JFS, et al. Modifications of arbovirus transmission in relation to construction of dams in Brazilian Amazonia Journal of the Brazilian Association for the Advancement of Science. 1992;44.

15. Taylor RM. Catalogue of arthropod-borne viruses of the world: a collection of data on registered arthropod-borne animal viruses: US Public Health Service; 1967.

16. Pinheiro FP, Bensabath G, Andrade AH, Lins ZC, Fraihi H, Tang AT, et al. Infectious diseases along Brazil's Trans-Amazon Highway: surveillance and research. Bull Pan Am Health Organ. 1974;8(111).

17. Srihongse S, Galindo P, Eldridge BF. A survey to assess potential human disease hazards along proposed sea level canal routes in Panama and Colombia. V. Arbovirus infection in non human vertebrates. Mil Med. 1974;139(6):449-53.

18. Nunes MR, Barbosa TF, Casseb LM, Nunes Neto JP, Segura Nde O, Monteiro HA, et al. Eco-epidemiologia dos arbovirus na area de influencia da rodovia Cuiaba-Santarem (BR 163), Estado do Para, Brasil. Cad Saude Publica. 2009;25(12):2583-602. Epub 2010/03/02. doi: 10.1590/s0102-311x2009001200006. PubMed PMID: 20191150.

19. Sanmartín C, Mackenzie RB, Trapido H, Barreto P, Mullenax CH, Gutiérrez E, et al. Encefalitis equina venezolana en Colombia, 1967. Bol Oficina Sanit Panam. 1973;74(2):108-37. Epub 1973/02/01. PubMed PMID: 4265714.

20. Galindo P, Srihongse S, De Rodaniche E, Grayson MA. An ecological survey for arboviruses in Almirante, Panama, 1959-1962. Am J Trop Med Hyg. 1966;15(3):385-400. Epub 1966/05/01. doi: 10.4269/ajtmh.1966.15.385. PubMed PMID: 4380043.

21. Cruz ACR, Prazeres AdSCd, Gama EC, Lima MFd, Azevedo RdSS, Casseb LMN, et al. Vigilância sorológica para arbovírus em Juruti, Pará, Brasil. Cadernos de saude publica. 2009;25(11):2517-23.

22. Price JL. Serological evidence of infection of Tacaribe virus and arboviruses in Trinidadian bats. Am J Trop Med Hyg. 1978;27(1 Pt 1):162-7. Epub 1978/01/01. doi: 10.4269/ajtmh.1978.27.162. PubMed PMID: 204207.

23. Pauvolid-Correa A, Juliano RS, Campos Z, Velez J, Nogueira RM, Komar N. Neutralising antibodies for Mayaro virus in Pantanal, Brazil. Mem Inst Oswaldo Cruz. 2015;110(1):125-33. Epub 2015/03/06. doi: 10.1590/0074-02760140383. PubMed PMID: 25742272; PubMed Central PMCID: PMCPMC4371226.

24. Medlin S, Deardorff ER, Hanley CS, Vergneau-Grosset C, Siudak-Campfield A, Dallwig R, et al. Serosurvey of Selected Arboviral Pathogens in Free-Ranging, Two-Toed Sloths (Choloepus Hoffmanni) and Three-Toed Sloths (Bradypus Variegatus) In Costa Rica, 2005-07. J Wildl Dis. 2016;52(4):883-92. Epub 2016/08/02. doi: 10.7589/2015-02-040. PubMed PMID: 27479900; PubMed Central PMCID: PMCPMC5189659.
