# Supplementary material for: A systematic review and meta-analysis of the potential non-human animal reservoirs and arthropod vectors of the Mayaro virus
Source: PLoS Negl Trop Dis. 2021 Dec 13;15(12):e0010016. doi: 10.1371/journal.pntd.0010016 (PMC8699665; doi:10.1371/journal.pntd.0010016)
Supplement: S2 Table — Includes all positive samples regardless of test method. (DOCX) [file pntd.0010016.s003.docx]

**S2 Table. MAYV positivity by taxa of wild birds in included studies**

| **Family** | **Genus** | **Species** | **Common Name** | **MAYV Positive Samples** | **Total Positive** | **Total Tested^#^** | **Country of study*** |
| --- | --- | --- | --- | --- | --- | --- | --- |
| ***Order: Passeriformes*** | | | | | | | |
| Bombycillidae | *Bombycilla* | *B. cedrorum* | Cedar waxwing | No | 0 | 1 | USA [1] |
| Cardinalidae | *Cyanocompsa* | *C. cyanoides* | Blue-black grosbeak | No | 0 | 2 | Colombia [2],  Brazil [3] |
|  | *Guiraca* | *G. caerulea* | Blue grosbeak | No | 0 | 8 | USA [1] |
|  | *Habia* | *H. rubica* | Red-crowned ant tanager | No | 0 | NA | Brazil [3] |
|  | *Passerina* | *P. ciris* | Painted bunting | No | 0 | 12 | USA [1] |
|  |  | *P. cyanea* | Indigo bunting | No | 0 | 60 | USA [1] |
|  | *Pheucticus* | *P. ludovicianus* | Rose-breasted grosbeak | No | 0 | 52 | USA [1],  Colombia [2] |
|  | *Piranga* | *P.* *olivacea* | Scarlet tanager | No | 0 | 70 | USA [1] |
|  |  | *P. rubra* | Summer tanager | No | 0 | 110 | USA [1] |
|  | *Richmondena* | *R. cardinalis* | Northern cardinal | No | 0 | 8 | USA [1] |
| Conopophagidae | *Conopophaga* | *C. roberti* | Hooded gnateater | No | 0 | 1 | Brazil [4] |
|  |  | *C. aurita* | Chestnut-belted gnateater | No | 0 | NA | Brazil [3] |
| Dendrocolaptidae | *Hylexetastes* | *H. perrotii* | Red-billed woodcreeper | No | 0 | NA | Brazil [3] |
|  | *Xiphorhynchus* | *X. picus* | Straight-billed Woodcreeper | No | 0 | NA | Brazil [3] |
|  | NA | NA | NA | Yes | 1 | 97 | Brazil **[5]*** |
| Formicariidae | *Formicarius* | *F. analis* | Black-faced antthrush | No | 0 | NA | Brazil [3] |
|  | NA | NA | NA | Yes | 5 | 444 | Brazil **[5]*** |
| Fringillidae | *Cyanocompsa* | *C. cyanoides* | Blue-black grosbeak | No | 0 | 2 | Colombia [2] |
|  | *Spinus* | *S. psaltria* | Lesser goldfinch | No | 0 | 13 | Colombia [2] |
|  |  | NA | NA | No | 0 | 2 | Colombia [2] |
|  | *Tiaris* | NA | NA | No | 0 | 1 | Colombia [2] |
|  | NA | NA | NA | Yes | 6 | 131 | Brazil **[5]*** |
| Furnariidae | *Automolus* | *A. infuscatus* | Olive-backed foliage-gleaner | No | 0 | NA | Brazil [3] |
|  |  | *A. rufipileatus* | Chestnut-crowned Foliage-gleaner | No | 0 | NA | Brazil [3] |
|  | *Deconychura* | *D. longicauda* | Long-tailed woodcreeper | No | 0 | NA | Brazil [3] |
|  | *Dendrocincla* | *D. fuliginosa* | Plain-brown woodcreeper | No | 0 | NA | Brazil [3] |
|  | *Glyphorynchus* | *G. spirurus* | Wedge-billed woodcreeper | No | 0 | NA | Brazil [3] |
|  | *Philydor* | *P. pyrrhodes* | Cinnamon-rumped foliage-gleaner | No | 0 | NA | Brazil [3] |
|  | *Sclerurus* | *S. mexicanus* | Tawny-throated leaftosser | No | 0 | NA | Brazil [3] |
|  | *Synallaxis* | *S. albescens* | Pale-breasted spinetail | No | 0 | 1 | Colombia [2] |
|  |  | *S. gujanensis* | Plain-crowned Spinetail | No | 0 | NA | Brazil [3] |
|  | *Xiphorhynchus* | *X. ocellatus* | Ocellated woodcreeper | No | 0 | 1 | Brazil [4] |
|  |  | *X. spixii* | Spix's woodcreeper | No | 0 | NA | Brazil [3] |
| Grallariidae | *Myrmothera* | *M. campanisona* | Thrush-like antpitta | No | 0 | 1 | Brazil [4] |
| Hirundinidae | *Hirundo* | *H. rustica* | Barn swallow | No | 0 | 4 | USA [1] |
|  | *Notiochelidon* | *N. cyanoleuca* | Blue-and-white swallow | No | 0 | 3 | Colombia [2] |
|  | *Stelgidopteryx* | *S. ruficollis* | Southern rough-winged swallow | No | 0 | 1 | Colombia [2] |
| Icteridae | *Agelaius* | *A. icterocephalus* | Yellow-hooded blackbird | No | 0 | 3 | Colombia [2] |
|  |  | *A. phoeniceus* | Red-winged blackbird | No | 0 | 65 | USA [1] |
|  | *Cacicus* | *C. cela* | Yellow-rumped cacique | No | 0 | NA | Brazil [3] |
|  | *Dolichonyx* | *D. orizivorus* | Bobolink | No | 0 | 8 | USA [1] |
|  | *Icterus* | *I. galbula* | Baltimore oriole | No | 0 | 2 | USA [1] |
|  |  | *I. prosthemelas* | Black-cowled oriole | No | 0 | 2 | Panama [6] |
|  |  | *I. spurius* | Orchard oriole | Yes | 1 | 223 | USA **[1]*** |
| Mimidae | *Dumetella* | *D. carolinensis* | Gray catbird | No | 0 | 134 | USA [1] |
| Motacillidae | *Anthus* | *A. hellmayri* | Hellmayr’s pippit | No | 0 | NA | Brazil [7] |
| Paridae | *Parus* | *P. bicolor* | Tufted titmouse | No | 0 | 1 | USA [1] |
| Parulidae | *Dendroica* | *D. striata* | Blackpoll warbler | No | 0 | 10 | USA [1] |
|  |  | *D. magnolia* | Magnolia warbler | No | 0 | 4 | USA [1] |
|  |  | *D. petechia* | American yellow warbler | No | 0 | 4 | USA [1] |
|  | *Geothlypis* | *G. aequinoctialis* | Masked yellowthroat | No | 0 | NA | Brazil [3] |
|  |  | *G. semiflava* | Olive-crowned yellowthroat | No | 0 | 1 | Colombia [2] |
|  |  | *G. trichas* | Common yellowthroat | No | 0 | 2 | USA [1] |
|  | *Helmitheros* | *H. vermivorus* | Worm-eating warbler | No | 0 | 5 | USA [1] |
|  | *Limnothlypis* | *L. swainsonii* | Swainson's warbler | No | 0 | 2 | USA [1] |
|  | *Mniotilta* | *M. varia* | Black-and-white warbler | No | 0 | 5 | USA [1] |
|  | *Oporornis* | *O. formosus* | Kentucky warbler | No | 0 | 6 | USA [1] |
|  |  | *O. philadelphiae* | Mourning warbler | No | 0 | 1 | Colombia [2] |
|  | *Protonotaria* | *P. citrea* | Prothonotary warbler | No | 0 | 36 | USA [1] |
|  | *Seiurus* | *S. aurocapillus* | Ovenbird | No | 0 | 31 | USA [1] |
|  |  | *S. motacilla* | Louisiana waterthrush | No | 0 | 1 | USA [1] |
|  |  | *S. noveboracensis* | Northern waterthrush | No | 0 | 35 | USA [1] |
|  | *Setophaga* | *S. ruticilla* | American redstart | No | 0 | 12 | USA [1] |
|  | *Vermivora* | *V. peregrina* | Tennessee warbler | No | 0 | 1 | USA [1] |
|  |  | *V. pinus* | Blue-winged warbler | No | 0 | 2 | USA [1] |
|  | *Wilsonia* | *W. citrina* | Hooded warbler | No | 0 | 13 | USA [1] |
| Passerellidae | *Arremon* | *A. tactiturnus* | Pectoral sparrow | Yes | 1 | NA | Brazil **[8]*** [3] |
|  | *Zonotrichia* | *Z. albicollis* | White-throated sparrow | No | 0 | 8 | USA [1] |
|  |  | *Z. capensis* | Rufous-collared sparrow | No | 0 | 20 | Colombia [2] |
| Pipridae | *Pipra* | *P. fasciicauda* | Band-tailed manakin | No | 0 | NA | Brazil [3] |
|  |  | *P. rubrocapilla* | Red-headed manakin | No | 0 | NA | Brazil [3] |
|  | NA | NA | NA | Yes | 1 | 229 | Brazil **[5]*** |
| Thamnophilidae | *Cercomacra* | *C. tyrannina* | Dusky antbird | Yes | 1 | N/A | Brazil **[8]*** |
|  |  | *C. nigrescens* | Blackish antbird | No | 0 | NA | Brazil [3] |
|  | *Formicivora* | *F. grisea* | Southern white-fringed antwren | Yes | 1 | NA | Brazil **[8]*** |
|  | *Hylophylax* | *H. naevia* | Spot-backed antbird | No | 0 | NA | Brazil [3] |
|  | *Hypocnemis* | *H. cantator* | Guianan warbling antbird | No | 0 | NA | Brazil [3] |
|  | *Phlegopsis* | *P. nigromaculata* | Black-spotted bare-eye | No | 0 | NA | Brazil [3] |
|  | *Percnostola* | *P. rufifrons* | Black-headed antbird | No | 0 | NA | Brazil [3] |
|  | *Pygiptila* | *P. stellaris* | Spot-winged antshrike | No | 0 | NA | Brazil [3] |
|  | *Pyriglena* | *P. leuconota* | East Amazonian fire-eye | No | 0 | NA | Brazil [3] |
|  | *Taraba* | *T. major* | Great antshrike | No | 0 | NA | Brazil [3] |
|  | *Thamnomanes* | *T. caesius* | Cinereous antshrike | No | 0 | NA | Brazil [3] |
|  | *Thamnophilus* | *T. aethiops* | White-shouldered antshrike | No | 0 | 1 | Brazil [3, 4] |
|  |  | *T. amazonicus* | Amazonian antshrike | No | 0 | NA | Brazil [3] |
| Thraupidae | *Coereba* | *C. flaveola* | Bananaquit | No | 0 | 2 | Colombia [2] |
|  | *Oryzoborus* | *O. angolensis* | Chestnut-bellied seed finch | No | 0 | NA | Brazil [3] |
|  | *Saltator* | *S. albicollis* | Lesser Antillean saltator | No | 0 | 7 | Colombia [2] |
|  |  | *S. coerulescens* | Greyish saltator | No | 0 | NA | Brazil [3] |
|  |  | *S. maximus* | Buff-throated saltator | No | 0 | NA | Brazil [3] |
|  | *Sicalis* | *S. luteola* | Grassland yellow finch | No | 0 | NA | Brazil [7] |
|  | *Ramphocelus* | *R. carbo* | Silver-beaked tanager | No | 0 | NA | Brazil [3] |
|  |  | *R. dimidiatus* | Crimson-backed tanager | No | 0 | 1 | Colombia [2] |
|  |  | *R. paserinii* | Scarlet-rumped tanager | No | 0 | 201 | Panama [6] |
|  | *Sporophila* | *S. caerulescens* | Double-collared seedeater | No | 0 | NA | Brazil [3] |
|  |  | *S. intermedia* | Grey seedeater | No | 0 | 11 | Colombia [2] |
|  |  | *S. minuta* | Ruddy-breasted seedeater | No | 0 | 5 | Colombia [2] |
|  |  | *S. nigricollis* | Yellow-bellied seedeater | No | 0 | 5 | Colombia [2] |
|  |  | NA | NA | No | 0 | 7 | Colombia [2] |
|  | *Tachyphonus* | *T. rufus* | White-lined tanager | No | 0 | NA | Brazil [3] |
|  | *Thraupis* | *T. episcopus* | Blue-gray tanager | No | 0 | 6 | Colombia [2],  Brazil [3] |
|  |  | *T. palmarum* | Palm tanager | No | 0 | NA | Brazil [3] |
|  | *Volatinia* | *V. jacarina* | Blue-black grassquit | No | 0 | 1 | Colombia [2] |
| Tityridae | *Onychorhynchus* | *O. coronatus* | Amazonian Royal Flycatcher | No | 0 | NA | Brazil [3] |
|  | *Schiffornis* | *S. turdina* | Brown-winged schiffornis | No | 0 | 1 | Brazil [4] |
| Troglodytidae | *Microcerculus* | *M. marginatus* | Scaly-breasted Wren | No | 0 | NA | Brazil [3] |
|  | *Thryothorus* | *T. genibarbis* | Moustached wren | No | 0 | NA | Brazil [3] |
|  |  | *T. ludovicianus* | Carolina wren | No | 0 | 1 | USA [1] |
|  | *Troglodytes* | *T. aedon* | House wren | No | 0 | 3 | Colombia [2] |
| Turdidae | *Catharus* | *C. ustulatus* | Swainson's thrush | No | 0 | 12 | Colombia [2],  USA [1] |
|  | *Hylocichla* | *H. fuscescens* | Veery | No | 0 | 30 | USA [1] |
|  |  | *H. minima* | Gray-cheeked thrush | No | 0 | 43 | USA [1] |
|  |  | *H. mustellina* | Wood thrush | No | 0 | 16 | USA [1] |
|  | *Turdus* | *T. fumigatus* | Cocoa thrush | No | 0 | NA | Brazil [3] |
|  |  | *T. grayi* | Clay-colored thrush | No | 0 | 43 | Panama [6] |
|  |  | *T. ignobilis* | Black-billed thrush | No | 0 | 22 | Colombia [2] |
|  |  | NA | NA | No | 0 | 2 | Colombia [2] |
| Tyrannidae | *Attila* | *A. spadiceus* | Bright-rumped attila | No | 0 | NA | Brazil [3] |
|  | *Contopus* | *C. virens* | Eastern wood pewee | No | 0 | 2 | USA [1] |
|  | *Elaenia* | *E. flavogaster* | Yellow-bellied elaenia | No | 0 | 10 | Colombia [2] |
|  | *Empidonax* | *E. virescens* | Acadian flycatcher | No | 0 | 5 | USA [1] |
|  | *Fluvicola* | *F. pica* | Pied water tyrant | No | 0 | 2 | Colombia [2] |
|  | *Mecocerculus* | *M. leucophrys* | White-throated tyrannulet | No | 0 | NA | Brazil [3] |
|  | *Myiozetetes* | *M. crinitus* | Great crested flycatcher | No | 0 | 11 | USA [1] |
|  |  | *M. granadensis* | Grey-capped flycatcher | No | 0 | 16 | Panama [6] |
|  |  | *M. similis* | Social flycatcher | No | 0 | 17 | Panama [6] |
|  |  | *M. cayanensis* | Rusty-margined flycatcher | No | 0 | 4 | Colombia [2] |
|  |  | NA | NA | No | 0 | 21 | Panama [6] |
|  | *Pitangus* | *P. sulphuratus* | Great kiskadee | No | 0 | 7 | Brazil [9], Colombia [2] |
|  | *Pyrocephalus* | *P. rubinus* | Scarlet flycatcher | No | 0 | 1 | Colombia [2] |
|  | *Todirostrum* | *T. cinereum* | Common tody-flycatcher | No | 0 | 1 | Colombia [2] |
|  |  | NA | NA | No | 0 | 1 | Colombia [2] |
|  | *Tyrannus* | *T. melancholicus* | Tropical kingbird | Yes | 1 | 4 | Brazil **[8]*** [9]**,** Colombia [2] |
|  |  | *T. tyrannus* | Eastern kingbird | No | 0 | 29 | USA [1] |
|  | NA | NA | NA | Yes | 1 | 103 | Brazil **[5]*,**  Colombia [2] |
| Vireonidae | *Vireo* | *V. altiloquus* | Black-whiskered Vireo | No | 0 | 3 | USA [1] |
|  |  | *V. flavifrons* | Yellow-throated vireo | No | 0 | 4 | USA [1] |
|  |  | *V. griseus* | White-eyed vireo | No | 0 | 4 | USA [1] |
|  |  | *V. olivaceus* | Red-eyed vireo | No | 0 | 158 | Colombia [2],  USA [1] |
| ***Order Pelecaniformes*** | | | | | | | |
| Ardeidae | *Bubulcus* | *B. ibis* | Cattle egret | No | 0 | 1 | Colombia [2] |
|  | *Butorides* | *B. virescens* | Green heron | No | 0 | 20 | Panama [6] |
|  | *Egretta* | *E. caerulea* | Little blue heron | No | 0 | 14 | Panama [6] |
|  | *Tigrisoma* | *T. lineatum* | Rufescent tiger heron | No | 0 | 1 | Peru [10] |
| ***Order Piciformes*** | | | | | | | |
| Bucconidae | *Malacoptila* | *M. rufa* | Rufous-necked puffbird | No | 0 | NA | Brazil [3] |
| Picidae | *Chrysoptilus* | *C. punctigula* | Spot-breasted woodpecker | No | 0 | 1 | Colombia [2] |
|  | *Dendrocopos* | *D. villosus* | Hairy Woodpecker | No | 0 | 3 | USA [1] |
|  | *Dryocopus* | *D. lineatus* | Lineated woodpecker | No | 0 | NA | Brazil [3] |
|  | *Picummus* | NA | NA | No | 0 | 1 | Colombia [2] |
|  | *Sphyrapicus* | *S. varius* | Yellow-bellied sapsucker | No | 0 | 3 | USA [1] |
| Ramphastidae | *Ramphastos* | *R. sulfuratus* | Keel-billed toucan | No | 0 | 28 | Panama [6] |
| ***Order: Charadriiformes*** | | | | | | | |
| Charadriidae | *Charadrius* | *C. collaris* | Collared plover | No | 0 | 8 | Brazil [9] |
|  |  | *C. semipalmatus* | Semipalmated plover | No | 0 | 3 | Brazil [7, 9] |
|  |  | *C. wilsonia* | Wilson's plover | No | 0 | 1 | Brazil [9] |
|  | *Pluvialis* | *P. squatarola* | Grey plover | Yes | 1 | 4 | Brazil **[9]*** [7] |
| Hematopodidae | *Haematopus* | *H. palliatus* | American oystercatcher | Yes | 1 | 6 | Brazil **[11]*** |
| Jacanidae | *Jacana* | *J. jacana* | Wattled jacana | No | 0 | 6 | Colombia [2] |
| Laridae | *Rynchops* | *R.niger* | Black skimmer | No | 0 | 9 | Brazil [7, 9, 11] |
|  | *Sterna* | *S. eurygnatha* | Cayenne tern | Yes | 1 | 7 | Brazil **[11]*** [7] |
|  |  | *S. hirundo* | Common tern | Yes | 23 | 342 | Brazil **[11]*** [7] |
|  |  | *S. hirundinaceae* | South American tern | No | 0 | NA | Brazil [7] |
|  |  | *S. maxima* | Royal tern | Yes | 1 | 1 | Brazil **[11]*** |
|  |  | *S. nilotica* | Gull-billed tern | Yes | 1 | 1 | Brazil **[11]*** |
|  |  | *S. superciliaris* | Yellow-billed tern | Yes | 2 | 12 | Brazil **[11]*** [7, 9] |
|  |  | *S. trudeaui* | Snowy-crowned tern | Yes | 12 | 56 | Brazil **[11]*** [7] |
| Recurvirostridae | *Himantopus* | *H. himantopus* | Black-winged stilt | No | 0 | NA | Brazil [7] |
| Scolopacidae | *Actitis* | *A. macularius* | Spotted sandpiper | Yes | 2 | 26 | Brazil **[9]*,**  USA [1],  Colombia [2] |
|  | *Arenaria* | *A. interpres* | Ruddy turnstone | Yes | 8 | 36 | Brazil **[11]*** [7, 9] |
|  | *Calidris* | *C. alba* | Sanderling | No | 0 | NA | Brazil [7] |
|  |  | *C. canutus* | Red knot | Yes | 7 | 54 | Brazil **[11]*** [7, 9] |
|  |  | *C. fuscicollis* | White-rumped sandpiper | Yes | 1 | 11 | Brazil **[11]*** [7] |
|  |  | *C. minutilla* | Least sandpiper | Yes | 1 | 6 | Brazil **[9]*** |
|  |  | *C. pusilla* | Semipalmated sandpiper | Yes | 1 | 30 | Brazil **[9]*** |
|  | *Limosa* | *L. haemastica* | Hudsonian godwit | Yes | 5 | 17 | Brazil **[11]*** [7] |
|  | *Tringa* | *T. flavipes* | Lesser yellowlegs | Yes | 4 | 5 | Brazil **[11]*** [7] |
|  |  | *T. melanoleuca* | Greater yellowlegs | No | 0 | 1 | Brazil [7, 9] |
|  |  | *T. solitaria* | Solitary sandpiper | No | 0 | 1 | Colombia [2] |
| ***Order Caprimulgiformes*** | | | | | | | |
| Caprimulgidae | *Caprimulgus* | NA | NA | No | 0 | 1 | Colombia [2] |
|  | NA | NA | NA | Yes | 1 | 5 | Brazil **[5]*** |
| ***Order Columbiformes*** | | | | | | | |
| Columbidae | *Geotrygon* | *G. montana* | Ruddy quail-dove | No | 0 | 1 | Brazil [4] [3] |
|  |  | *G. violacea* | Violaceous quail-dove | No | 0 | NA | Brazil [3] |
|  | *Columbina* | *C. talpacoti* | Ruddy ground dove | No | 0 | 6 | Colombia [2] |
|  |  | *C. passerina* | Common ground dove | No | 0 | 6 | Colombia [2], Brazil [3] |
|  | *Leptotila* | *L. rufaxilla* | Grey-fronted dove | No | 0 | NA | Brazil [3] |
|  |  | *L. plumbeiceps* | Grey-headed dove | No | 0 | 5 | Colombia [2] |
|  |  | NA | NA | No | 0 | 2 | Colombia [2] |
|  | *Columbigallina* | NA | NA | Yes | 34 | 121 | Brazil **[12]***,  Colombia [2] |
|  | NA | NA | NA | Yes | 1 | 34 | Brazil **[5]*** |
| ***Order Coraciiformes*** | | | | | | | |
| Momotidae | *Momotus* | *M. momota* | Amazonian motmot | No | 0 | NA | Brazil [3] |
| Alcedinidae | *Chloroceryle* | *C. inda* | Green-and-rufous kingfisher | No | 0 | NA | Brazil [3] |
| ***Order Podicipediformes*** | | | | | | | |
| Podicipedidae | *Podiceps* | *P. dominicus* | Least grebe | No | 0 | 2 | Colombia [2] |
|  |  | *P. major* | Great grebe | No | 0 | NA | Brazil [7] |
| ***Order Psittaciformes*** | | | | | | | |
| Psittacidae | *Ara* | *A. ararauna* | Blue and yellow macaw | No | 0 | 2 | Peru [10] |
|  | *Forpus* | *F. conspicillatus* | Spectacled parrotlet | No | 0 | 1 | Colombia [2] |
| ***Order Cuculiformes*** | | | | | | | |
| Cuculidae | *Coccyzus* | *C. americanus* | Yellow-billed cuckoo | No | 0 | 40 | USA [1] |
|  |  | *C. erythropthalmus* | Black-billed cuckoo | No | 0 | 3 | USA [1] |
|  |  | *C. pumilus* | Dwarf cuckoo | No | 0 | 1 | Colombia [2] |
|  |  | NA | NA | No | 0 | 2 | Colombia [2] |
|  | *Crotophaga* | *C. ani* | Smooth-billed ani | No | 0 | 2 | Colombia [2] |
|  |  | *C. sulcirostris* | Groove-billed ani | No | 0 | 16 | Panama [6] |
| ***Order Galliformes*** | | | | | | | |
| Cracidae | *Ortalis* | *O. guttata* | Speckled chachalaca | No | 0 | 1 | Peru [10] |
| ***Order Strigiformes*** | | | | | | | |
| Strigidae | *Glaucidium* | *G. brasilianum* | Ferruginous pygmy owl | No | 0 | NA | Brazil [3] |
|  | *Otus* | *O. choliba* | Tropical screech owl | No | 0 | 3 | Colombia [2] |
| ***Order Apodiformes*** | | | | | | | |
| Trochilidae | *Gloucis* | *G. aenea* | Bronzy hermit | No | 0 | 1 | Colombia [2] |
|  | NA | NA | NA | No | 0 | 2 | Colombia [2] |
| ***Order Gruiformes*** | | | | | | | |
| Rallidae | *Gallinula* | *G. chloropus* | Common moorhen | No | 0 | 3 | Colombia [2] |
|  | *Rallus* | *R. longirostris* | Mangrove rail | No | 0 | 2 | Brazil [9] |
| ***Order Tinamiformes*** | | | | | | | |
| Tinamidae | *Tinamus* | *T. major* | Great tinamou | No | 0 | 2 | Peru [10] |

MAYV: Mayaro virus

^#^Total is pooled across all studies. A value of NA indicates that a study reported testing an animal for MAYV but did not specify how many were tested.

^*^Indicates the location where the positive animal was found and the citation for the study that reported the positive animal.

References

1. Calisher CH, Gutierrez E, Maness KS, Lord RD. Isolation of Mayaro virus from a migrating bird captured in Louisiana in 1967. Bull Pan Am Health Organ. 1974;8(3):243-8. Epub 1974/01/01. PubMed PMID: 4418030.

2. Sanmartín C, Mackenzie RB, Trapido H, Barreto P, Mullenax CH, Gutiérrez E, et al. Encefalitis equina venezolana en Colombia, 1967. Bol Oficina Sanit Panam. 1973;74(2):108-37. Epub 1973/02/01. PubMed PMID: 4265714.

3. Nunes MR, Barbosa TF, Casseb LM, Nunes Neto JP, Segura Nde O, Monteiro HA, et al. Eco-epidemiologia dos arbovirus na area de influencia da rodovia Cuiaba-Santarem (BR 163), Estado do Para, Brasil. Cad Saude Publica. 2009;25(12):2583-602. Epub 2010/03/02. doi: 10.1590/s0102-311x2009001200006. PubMed PMID: 20191150.

4. Cruz ACR, Prazeres AdSCd, Gama EC, Lima MFd, Azevedo RdSS, Casseb LMN, et al. Vigilância sorológica para arbovírus em Juruti, Pará, Brasil. Cadernos de saude publica. 2009;25(11):2517-23.

5. Hoch AL, Peterson NE, LeDuc JW, Pinheiro FP. An outbreak of Mayaro virus disease in Belterra, Brazil. III. Entomological and ecological studies. Am J Trop Med Hyg. 1981;30(3):689-98. Epub 1981/05/01. doi: 10.4269/ajtmh.1981.30.689. PubMed PMID: 6266265.

6. Galindo P, Srihongse S, De Rodaniche E, Grayson MA. An ecological survey for arboviruses in Almirante, Panama, 1959-1962. Am J Trop Med Hyg. 1966;15(3):385-400. Epub 1966/05/01. doi: 10.4269/ajtmh.1966.15.385. PubMed PMID: 4380043.

7. Araújo FAA, Vianna RdST, Andrade Filho GVd, Melhado DL, Todeschini B, Cavalcante e Cavalcanti G, et al. Segundo inquérito sorológico em aves migratórias e residentes do parque nacional da Lagoa do Peixe/RS para detecção do vírus da Febre da Febre do Nilo Ocidental e outros vírus. In: Ministério da Saúde Secretaria de Vigilância em Saúde, editor. Boletim Eletrônico Epidemiologico, 2004.

8. Degallier N, Travassos da Rosa AP, Vasconcelos PFC, Hervé JP, Sa Filho GC, Travassos da Rosa JFS, et al. Modifications of arbovirus transmission in relation to construction of dams in Brazilian Amazonia Journal of the Brazilian Association for the Advancement of Science. 1992;44.

9. Araujo FAA, Lima PC, Andrade MA, de Sá Jayme V, Ramos DG, Da Silveira SL. Soroprevalência de anticorpos “anti-arbovírus” de importância em saúde pública em aves selvagens, Brasil–2007 e 2008. Ciênc Anim Brasil. 2012;13(1):115-23. doi: 10.5216/cab.v13i1.16834.

10. Perez JG, Carrera JP, Serrano E, Pitti Y, Maguina JL, Mentaberre G, et al. Serologic Evidence of Zoonotic Alphaviruses in Humans from an Indigenous Community in the Peruvian Amazon. Am J Trop Med Hyg. 2019. Epub 2019/10/02. doi: 10.4269/ajtmh.18-0850. PubMed PMID: 31571566.

11. Araujo FAA, Wada MY, da Silva EV, Cavalcante GC, Magalhaes VS, de Andrade Filho GV, et al. Primeiro inquérito sorológico em aves migratórias e nativas do Parque Nacional da Lagoa do Peixe/RS para detecção do vírus do Nilo Ocidental. In: Ministério da Saúde Secretaria de Vigilância em Saúde, editor. Boletim Eletrônico Epidemiologico, 2003.

12. Taylor RM. Catalogue of arthropod-borne viruses of the world: a collection of data on registered arthropod-borne animal viruses: US Public Health Service; 1967.
