# Supplementary material for: A systematic review and meta-analysis of the potential non-human animal reservoirs and arthropod vectors of the Mayaro virus
Source: PLoS Negl Trop Dis. 2021 Dec 13;15(12):e0010016. doi: 10.1371/journal.pntd.0010016 (PMC8699665; doi:10.1371/journal.pntd.0010016)
Supplement: S3 Table — (DOCX) [file pntd.0010016.s004.docx]

**S3 Table. MAYV positivity in domestic or sentinel animals studied**

| **Animal Type** | **MAYV positive Samples?** | **Total Positive** | **Total Tested^#^** | **Positivity Confirmed by NT?^%^** | **Country of study*** |
| --- | --- | --- | --- | --- | --- |
| Domestic Horse | Yes | 26 | 1096 | Yes | Brazil **[1]* [2]*** [3] |
|  |  | 51 | 859 | No | Brazil **[4]* [5]*** [6] |
| Domestic Cattle/Buffalo | Yes | 14 | 1103 | No | Brazil **[7]*,**  Colombia [8] |
| Domestic Dog | Yes | 2 | 22 | No | Brazil **[9]*** [6]  Colombia [8] |
| Sentinel Monkeys | Yes | 2 | 14 | No | Panama **[10]*** |
| Domestic Donkey | Yes | 1 | 24 | Yes | Brazil **[2]*** |
| Sentinel Hamster | Yes | 1 | NA | NA (RT-PCR) | Venezuela **[11]*** |
| Domestic Mule | No | 0 | 30 | NA | Brazil [2] |
| Domestic Unspecified Equid | No | 0 | 3 | NA | Brazil [2] |
| Domestic Sheep | No | 0^^^ | 622 | NA | Brazil [2,7] |
| Domestic Hen/Chicken | No | 0 | 67 | NA | Brazil [6,9,12]  Colombia [8] |
| Domestic Pig | No | 0 | 13 | NA | Brazil [6],  Colombia [8] |
| Domestic Duck | No | 0 | 11 | NA | Brazil [6,12] |
| Sentinel *Aotus nancymae* monkeys | No | 0 | 20 | NA | Peru [13] |

NT: Neutralization test; HI: hemagglutination inhibition; RT-PCR: reverse transcription polymerase chain reaction; MAYV: Mayaro virus

^%^*Yes* indicates that MAYV positivity was confirmed with an NT. *No* indicates that MAYV positivity was based on HI test only. *NA* indicates that no positivity was reported.

^#^Total is pooled across all studies. A value of NA indicates that a study reported testing an animal for MAYV but did not specify how many were tested.

^*^Indicates the location where the positive animal was found and the citation for the study that reported the positive animal.

^^^Neutralizing antibodies detected but did not meet the study’s diagnostic criteria.

References

1. Gomes FA, Jansen AM, Machado RZ, Jesus Pena HF, Fumagalli MJ, Silva A, et al. Serological evidence of arboviruses and coccidia infecting horses in the Amazonian region of Brazil. PloS One. 2019;14(12):e0225895. Epub 2019/12/13. doi: 10.1371/journal.pone.0225895. PubMed PMID: 31830142.

2. Pauvolid-Correa A, Juliano RS, Campos Z, Velez J, Nogueira RM, Komar N. Neutralising antibodies for Mayaro virus in Pantanal, Brazil. Mem Inst Oswaldo Cruz. 2015;110(1):125-33. Epub 2015/03/06. doi: 10.1590/0074-02760140383. PubMed PMID: 25742272; PubMed Central PMCID: PMCPMC4371226.

3. Pauvolid-Correa A, Tavares FN, Costa EV, Burlandy FM, Murta M, Pellegrin AO, et al. Serologic evidence of the recent circulation of Saint Louis encephalitis virus and high prevalence of equine encephalitis viruses in horses in the Nhecolandia sub-region in South Pantanal, Central-West Brazil. Mem Inst Oswaldo Cruz. 2010;105(6):829-33. Epub 2010/10/15. doi: 10.1590/s0074-02762010000600017. PubMed PMID: 20945001.

4. Araujo FAA, Andrade MA, Jayme VS, Santos AL, Roman APM, Ramos DG, et al. Anticorpos antialfavírus detectados em equinos durante diferentes epizootias de encefalite equina, Paraíba, 2009. Rev Bras Ciênc Vet. 2012;19(1):80-5. doi: 10.4322/rbcv.2014.086.

5. Casseb AdR, Brito TC, Silva MRMd, Chiang JO, Martins LC, Silva SPd, et al. Prevalence of antibodies to equine alphaviruses in the State of Pará, Brazil. Arq Inst Biol. 2016;83. doi: 10.1590/1808-1657000202014.

6. Araújo FAA, Vianna RdST, Andrade Filho GVd, Melhado DL, Todeschini B, Cavalcante e Cavalcanti G, et al. Segundo inquérito sorológico em aves migratórias e residentes do parque nacional da Lagoa do Peixe/RS para detecção do vírus da Febre da Febre do Nilo Ocidental e outros vírus. In: Ministério da Saúde Secretaria de Vigilância em Saúde, editor. Boletim Eletrônico Epidemiologico, 2004.

7. Casseb AdR. Soroprevalência de anticorpos e padronização do teste ELISA sanduíche indireto para 19 tipos de arbovírus em herbívoros domésticos [Ph.D. Thesis]. Belém: Universidade Federal do Pará; 2010. Available from: <http://repositorio.ufpa.br/jspui/handle/2011/4760>.

8. Sanmartín C, Mackenzie RB, Trapido H, Barreto P, Mullenax CH, Gutiérrez E, et al. Encefalitis equina venezolana en Colombia, 1967. Bol Oficina Sanit Panam. 1973;74(2):108-37. Epub 1973/02/01. PubMed PMID: 4265714.

9. Araujo FAA, Wada MY, da Silva EV, Cavalcante GC, Magalhaes VS, de Andrade Filho GV, et al. Primeiro inquérito sorológico em aves migratórias e nativas do Parque Nacional da Lagoa do Peixe/RS para detecção do vírus do Nilo Ocidental. In: Ministério da Saúde Secretaria de Vigilância em Saúde, editor. Boletim Eletrônico Epidemiologico, 2003.

10. Srihongse S, Galindo P, Eldridge BF. A survey to assess potential human disease hazards along proposed sea level canal routes in Panama and Colombia. V. Arbovirus infection in non human vertebrates. Mil Med. 1974;139(6):449-53.

11. Medina G, Garzaro DJ, Barrios M, Auguste AJ, Weaver SC, Pujol FH. Genetic diversity of Venezuelan alphaviruses and circulation of a Venezuelan equine encephalitis virus subtype IAB strain during an interepizootic period. Am J Trop Med Hyg. 2015;93(1):7-10. Epub 2015/05/06. doi: 10.4269/ajtmh.14-0543. PubMed PMID: 25940191; PubMed Central PMCID: PMCPMC4497907.

12. Hoch AL, Peterson NE, LeDuc JW, Pinheiro FP. An outbreak of Mayaro virus disease in Belterra, Brazil. III. Entomological and ecological studies. Am J Trop Med Hyg. 1981;30(3):689-98. Epub 1981/05/01. doi: 10.4269/ajtmh.1981.30.689. PubMed PMID: 6266265.

13. Turell MJ, Gozalo AS, Guevara C, Schoeler GB, Carbajal F, Lopez-Sifuentes VM, et al. Lack of Evidence of Sylvatic Transmission of Dengue Viruses in the Amazon Rainforest Near Iquitos, Peru. Vector Borne Zoonotic Dis. 2019;19(9):685-9. Epub 2019/04/10. doi: 10.1089/vbz.2018.2408. PubMed PMID: 30964397; PubMed Central PMCID: PMCPMC6716187.
