# Supplementary material for: A systematic review and meta-analysis of the potential non-human animal reservoirs and arthropod vectors of the Mayaro virus
Source: PLoS Negl Trop Dis. 2021 Dec 13;15(12):e0010016. doi: 10.1371/journal.pntd.0010016 (PMC8699665; doi:10.1371/journal.pntd.0010016)
Supplement: S5 Table — (DOCX) [file pntd.0010016.s006.docx]

**S5 Table. Primate genera pooled prevalence table (random effects with Freeman-Tukey double arcsine transformation)**

| **Primate Genus** | **Positives Included^1^** | **Studies (n)** | **Total (n)** | **Positive (n)** | **Pooled Prev. (%)** | **95% CI** | **I^2^ (%)** | ***τ*^2^** | **P-value** |
| --- | --- | --- | --- | --- | --- | --- | --- | --- | --- |
| *Cebus/Sapajus* | HI and NT | 9 | 316 | 25 | 3.7 | 0.0; 11.1 | 61 | 0.0132 | <0.01 |
|  | NT only | 9 | 293 | 2 | 0.0 | 0.0; 0.0 | 13 | 0.0014 | 0.32 |
| *Alouatta* | HI and NT | 8 | 213 | 63 | 32.2 | 0.0; 79.2 | 95 | 0.2257 | <0.01 |
|  | NT only | 8 | 206 | 56 | 20.8 | 0.0; 68.9 | 94 | 0.2263 | <0.01 |
| *Callithrix* | HI and NT | 3 | 123 | 32 | 17.8 | 8.6; 28.5 | 0 | 0 | 0.54 |
|  | NT only | 3 | 123 | 32 | 17.8 | 8.6; 28.5 | 0 | 0 | 0.54 |
| *Saguinus* | HI and NT | 2 | 74 | 8 | 6.3 | 0.0; 35.5 | 90 | 0.0628 | <0.01 |
|  | NT only | 2 | 74 | 8 | 6.3 | 0.0; 35.5 | 90 | 0.0628 | <0.01 |
| *Lagothrix* | HI and NT | 1 | 11 | 6 | 54.5 | 24.2; 83.3 | NA | NA | NA |
|  | NT only | 1 | 11 | 6 | 54.5 | 24.2; 83.3 | NA | NA | NA |
| *Saimiri* | HI and NT | 3 | 10 | 5 | 45.9 | 0.0; 100.0 | 62 | 0.1254 | 0.07 |
|  | NT only | 2 | 9 | 4 | 30.3 | 0.0; 98.5 | 76 | 0.1696 | 0.04 |
| *Aotus* | HI and NT | 2 | 10 | 1 | 6.3 | 0.0; 43.6 | 35 | 0.0253 | 0.21 |
|  | NT only | 2 | 9 | 0 | 0.0 | 0.0; 20.0 | 0 | 0 | 0.84 |

MAYV: Mayaro virus; HI: hemagglutination inhibition; NT: neutralization test; CI: confidence interval

^1^ The first analysis (HI and NT) included all positive samples, regardless of test method. A sensitivity analysis was conducted that included only positive samples that were confirmed with NT.
