# Supplementary material for: A systematic review and meta-analysis of the potential non-human animal reservoirs and arthropod vectors of the Mayaro virus
Source: PLoS Negl Trop Dis. 2021 Dec 13;15(12):e0010016. doi: 10.1371/journal.pntd.0010016 (PMC8699665; doi:10.1371/journal.pntd.0010016)
Supplement: S6 Table — (DOCX) [file pntd.0010016.s007.docx]

**S6 Table. Primate genera pooled prevalence table (random effects using GLMM with logit transformation)**

| **Primate Genus** | **Positives Included^1^** | **Studies (n)** | **Total (n)** | **Positive (n)** | **Pooled Prev. (%)** | **95% CI** | **I^2^ (%)** | ***τ*^2^** | **P-value** |
| --- | --- | --- | --- | --- | --- | --- | --- | --- | --- |
| *Cebus/Sapajus* | HI and NT | 9 | 316 | 25 | 7.5 | 3.5; 15.3 | 59 | 0.6576 | 0.15 |
|  | NT only | 9 | 293 | 2 | 0.3 | 0.0; 9.0 | 72 | 4.7055 | 1.00 |
| *Alouatta* | HI and NT | 8 | 213 | 63 | 24.0 | 2.2; 81.6 | 94 | 10.4411 | 1.00 |
|  | NT only | 8 | 206 | 56 | 10.4 | 0.3; 79.7 | 94 | 16.7758 | 1.00 |
| *Callithrix* | HI and NT | 3 | 123 | 32 | 26.0 | 19.0; 34.5 | 0 | 0 | 1.00 |
|  | NT only | 3 | 123 | 32 | 26.0 | 19.0; 34.5 | 0 | 0 | 1.00 |
| *Saguinus* | HI and NT | 2 | 74 | 8 | 3.7 | 0.1; 63.4 | 77 | 3.7403 | 1.00 |
|  | NT only | 2 | 74 | 8 | 3.7 | 0.1; 63.4 | 77 | 3.7403 | 1.00 |
| *Lagothrix* | HI and NT | 1 | 11 | 6 | 54.5 | 26.8; 79.7 | NA | NA | NA |
|  | NT only | 1 | 11 | 6 | 54.5 | 26.8; 79.7 | NA | NA | NA |
| *Saimiri* | HI and NT | 3 | 10 | 5 | 47.7 | 8.9; 89.5 | 51 | 1.7960 | 1.00 |
|  | NT only | 2 | 9 | 4 | 31.1 | 1.6; 92.8 | 60 | 2.3144 | 1.00 |
| *Aotus* | HI and NT | 2 | 10 | 1 | 10.0 | 1.4; 46.7 | 0 | 0 | 1.00 |
|  | NT only | 2 | 9 | 0 | NA^2^ | NA | NA | NA | NA |

MAYV: Mayaro virus; GLMM: generalized linear mixed model; HI: hemagglutination inhibition; NT: neutralization test; CI: confidence interval

^1^ The first analysis (HI and NT) included all positive samples, regardless of test method. A sensitivity analysis was conducted that included only positive samples that were confirmed with NT.

^2^ Cannot fit model due to zero events.
