# Supplementary material for: A systematic review and meta-analysis of the potential non-human animal reservoirs and arthropod vectors of the Mayaro virus
Source: PLoS Negl Trop Dis. 2021 Dec 13;15(12):e0010016. doi: 10.1371/journal.pntd.0010016 (PMC8699665; doi:10.1371/journal.pntd.0010016)
Supplement: S7 Table — (DOCX) [file pntd.0010016.s008.docx]

**S7 Table. Pooled prevalence table (****fixed effects with Freeman-Tukey double arcsine transformation)**

| **Order** | **Positives Included^1^** | **Studies (n)** | **Total (n)** | **Positive (n)** | **Pooled Prev. (%)** | **95% CI** | **I^2^ (%)** | ***τ*^2^** | **P-value** |
| --- | --- | --- | --- | --- | --- | --- | --- | --- | --- |
| ***Mammals*** | | | | | | | | | |
| Primate | HI and NT | 13 | 897 | 153 | 11.9 | 9.8; 14.2 | 95 | 0.0692 | <0.01 |
|  | NT only | 13 | 858 | 114 | 6.5 | 4.8; 8.4 | 96 | 0.0851 | <0.01 |
| Pilosa | HI and NT | 7 | 297 | 15 | 0.0 | 0.0; 0.1 | 84 | 0.0338 | <0.01 |
|  | NT only | 7 | 296 | 14 | 0.0 | 0.0; 0.0 | 82 | 0.0305 | <0.01 |
| Rodentia | HI and NT | 7 | 1557 | 90 | 2.1 | 1.2; 3.1 | 91 | 0.0160 | <0.01 |
|  | NT only | 7 | 1486 | 19 | 0.0 | 0.0; 0.0 | 90 | 0.0153 | <0.01 |
| Domestic Equids | HI and NT | 6 | 1955 | 41 | 0.2 | 0.0; 0.6 | 90 | 0.0085 | <0.01 |
|  | NT only | 6 | 1940 | 26 | 0.0 | 0.0; 0.0 | 90 | 0.0087 | <0.01 |
| Didelphimorphia | HI and NT | 6 | 369 | 25 | 4.2 | 2.0; 6.9 | 68 | 0.0101 | <0.01 |
|  | NT only | 6 | 353 | 9 | 0.1 | 0.0; 1.3 | 74 | 0.0141 | <0.01 |
| Carnivora | HI and NT | 5 | 40 | 2 | 0.1 | 0.0; 8.1 | 0 | 0 | 0.71 |
|  | NT only | 5 | 40 | 2 | 0.1 | 0.0; 8.1 | 0 | 0 | 0.71 |
| Cingulata | HI and NT | 4 | 70 | 6 | 0.5 | 0.0; 7.4 | 35 | 0.0198 | 0.20 |
|  | NT only | 4 | 70 | 6 | 0.5 | 0.0; 7.4 | 35 | 0.0198 | 0.20 |
| Artiodactyla | HI and NT | 2 | 26 | 1 | 1.6 | 0.0; 12.7 | 46 | 0.0172 | 0.17 |
|  | NT only | 2 | 26 | 1 | 1.6 | 0.0; 12.7 | 46 | 0.0172 | 0.17 |
| ***Birds*^2^** | | | | | | | | | |
| Charadriiformes | HI and NT | 3 | 641 | 71 | 9.5 | 7.2; 12.2 | 61 | 0.0045 | 0.08 |
| Passeriformes | HI and NT | 4 | 1166 | 14 | 0.0 | 0.0; 0.0 | 27 | 0.0010 | 0.25 |
| Columbiformes | HI and NT | 4 | 171 | 35 | 11.7 | 5.6; 19.0 | 87 | 0.0591 | <0.01 |

MAYV: Mayaro virus; HI: hemagglutination inhibition; NT: neutralization test; CI: confidence interval

^1^ The first analysis included all positive samples, regardless of test method. The second analysis included only the positive samples that were confirmed with NT.

^2^ Only one study reporting MAYV positivity in birds used confirmatory NT.
