# Supplementary material for: A systematic review and meta-analysis of the potential non-human animal reservoirs and arthropod vectors of the Mayaro virus
Source: PLoS Negl Trop Dis. 2021 Dec 13;15(12):e0010016. doi: 10.1371/journal.pntd.0010016 (PMC8699665; doi:10.1371/journal.pntd.0010016)
Supplement: S8 Table — (DOCX) [file pntd.0010016.s009.docx]

**S8 Table. Pooled prevalence table (fixed effects using GLMM with logit transformation)**

| **Order** | **Positives Included^1^** | **Studies (n)** | **Total (n)** | **Positive (n)** | **Pooled Prev. (%)** | **95% CI** | **I^2^ (%)** | ***τ*^2^** | **P-value** |
| --- | --- | --- | --- | --- | --- | --- | --- | --- | --- |
| ***Mammals*** | | | | | | | | | |
| Primate | HI and NT | 13 | 897 | 153 | 17.1 | 14.7; 19.7 | 96 | 3.2572 | <0.01 |
|  | NT only | 13 | 858 | 114 | 13.3 | 11.2; 15.7 | 98 | 11.7937 | 0.05 |
| Pilosa | HI and NT | 7 | 297 | 15 | 5.1 | 3.1; 8.2 | 90 | 9.8670 | 1.00 |
|  | NT only | 7 | 296 | 14 | 4.7 | 2.8; 7.8 | 92 | 13.7165 | 1.00 |
| Rodentia | HI and NT | 7 | 1557 | 90 | 5.8 | 4.7; 7.1 | 93 | 2.6550 | 0.99 |
|  | NT only | 7 | 1486 | 19 | 1.3 | 0.8; 2.0 | 95 | 9.4525 | 0.99 |
| Domestic Equids | HI and NT | 6 | 1955 | 41 | 2.1 | 1.5; 2.8 | 92 | 2.0474 | <0.01 |
|  | NT only | 6 | 1940 | 26 | 1.3 | 0.9; 2.0 | 96 | 7.6678 | <0.01 |
| Didelphimorphia | HI and NT | 6 | 369 | 25 | 6.8 | 4.6; 9.8 | 85 | 2.1787 | 1.00 |
|  | NT only | 6 | 353 | 9 | 2.5 | 1.3; 4.8 | 90 | 10.4733 | 1.00 |
| Carnivora | HI and NT | 5 | 40 | 2 | 5.0 | 1.3; 17.9 | 3 | 0.0522 | 1.00 |
|  | NT only | 5 | 40 | 2 | 5.0 | 1.3; 17.9 | 3 | 0.0522 | 1.00 |
| Cingulata | HI and NT | 4 | 70 | 6 | 8.6 | 3.9; 17.8 | 34 | 0.5502 | 0.14 |
|  | NT only | 4 | 70 | 6 | 8.6 | 3.9; 17.8 | 34 | 0.5502 | 0.14 |
| Artiodactyla | HI and NT | 2 | 26 | 1 | 3.8 | 0.5; 22.8 | 0 | 0 | 1.00 |
|  | NT only | 2 | 26 | 1 | 3.8 | 0.5; 22.8 | 0 | 0 | 1.00 |
| ***Birds^28^*** | | | | | | | | | |
| Charadriiformes | HI and NT | 3 | 641 | 71 | 11.1 | 8.9; 13.7 | 29 | 0.0846 | 0.19 |
| Passeriformes | HI and NT | 4 | 1166 | 14 | 1.2 | 0.7; 2.0 | 0 | 0 | 1.00 |
| Columbiformes | HI and NT | 4 | 171 | 35 | 20.5 | 15.1; 27.2 | 74 | 2.4841` | 0.10 |

MAYV: Mayaro virus; HI: hemagglutination inhibition; NT: neutralization test; CI: confidence interval

^1^ The first analysis included all positive samples, regardless of test method. The second analysis included only the positive samples that were confirmed with NT.

^2^ Only one study reporting MAYV positivity in birds used confirmatory NT.
