# Supplementary material for: A systematic review and meta-analysis of the potential non-human animal reservoirs and arthropod vectors of the Mayaro virus
Source: PLoS Negl Trop Dis. 2021 Dec 13;15(12):e0010016. doi: 10.1371/journal.pntd.0010016 (PMC8699665; doi:10.1371/journal.pntd.0010016)
Supplement: S9 Table — (DOCX) [file pntd.0010016.s010.docx]

**S9 Table. Complete arthropod results by genus**

| **Study** | **Test Method** | **Genus** | **Total (n)** | **MAYV Detected?** |
| --- | --- | --- | --- | --- |
| Aitken, 1969 [1] | Virus isolation | *Acanthocera* | 1 | No |
|  |  | *Aedomyia* | 12 | No |
|  |  | *Aedes* | 367994 | No |
|  |  | *Amblyomma* | 6550 | No |
|  |  | *Anopheles* | 66073 | No |
|  |  | *Boophilus* | 1759 | No |
|  |  | *Chrysops* | 87 | No |
|  |  | *Culex* | 238771 | No |
|  |  | *Culicoides* | 10079 | No |
|  |  | *Deinocerites* | 15 | No |
|  |  | *Diachlorus* | 1078 | No |
|  |  | *Fahrenholzia* | 187 | No |
|  |  | *Gigantolaelaps* | 5312 | No |
|  |  | *Haemagogus* | 18561 | No |
|  |  | *Hoplopleura* | 2080 | No |
|  |  | *Ixodes* | 78 | No |
|  |  | *Leucotabanus* | 18 | No |
|  |  | *Limatus* | 50730 | No |
|  |  | *Mansonia* | 127963 | Yes |
|  |  | *Ornithodoros* | 1922 | No |
|  |  | *Orthopodomyia* | 19 | No |
|  |  | *Philornis* | 179 | No |
|  |  | *Phlebotomus* | 12841 | No |
|  |  | *Phoniomyia* | 63689 | No |
|  |  | *Polygenis* | 386 | No |
|  |  | *Psorophora* | 177136 | No |
|  |  | *Rhipicephalus* | 270 | No |
|  |  | *Sabethes* | 5059 | No |
|  |  | *Simulium* | 546 | No |
|  |  | *Stibasoma* | 35 | No |
|  |  | *Stomoxys* | 23 | No |
|  |  | *Xenopsylla* | 7 | No |
|  |  | *Tabanus* | 72 | No |
|  |  | *Trichoprosopon* | 20500 | No |
|  |  | *Uranotaenia* | 71 | No |
|  |  | *Wyeomyia* | 346093 | No |
| Azevedo, 2009 [2] | Virus isolation | *Haemagogus* | 188 | Yes |
|  |  | Other^a^ | 644 | No |
| Batista, 2012 [3] | Virus isolation | *Aedes* | 59 | No |
|  |  | *Culex* | 8 | No |
|  |  | *Haemagogus* | 11 | No |
|  |  | *Psorophora* | 9 | No |
|  |  | *Sabethes* | 35 | No |
| Carrera, 2020 [4] | RT-PCR | *Culex* | 113 | No |
| Catenacci, 2017 [5] | RT-PCR | *Aedes* | 1 | No |
|  |  | *Culex* | 3 | No |
|  |  | *Flebotominae* | 11 | No |
|  |  | *Haemagogus* | 17 | No |
|  |  | *Limatus* | 83 | No |
|  |  | *Mansonia* | 1 | No |
|  |  | *Psorophora* | 1 | No |
|  |  | *Sabethes* | 6 | No |
|  |  | *Runchomya* | 1 | No |
|  |  | *Wyeomyia* | 115 | Yes |
| Degallier, 1992 [6] | Virus isolation | NA | 2005069 | No |
| Esposito, 2015 [7] | Virus isolation | *Haemagogus* | NA | Yes |
| Ferreira, 2020 [8] | RT-PCR; Virus isolation | *Aedes* | 1139 | No |
|  |  | *Culex* | 9429 | Yes |
|  |  | *Psorophora* | 1 | No |
| Galindo, 1966 [9] | Virus isolation | *Aedes* | 50616 | No |
|  |  | *Anopheles* | 4515 | No |
|  |  | *Culex* | 166921 | No |
|  |  | *Mansonia* | 59693 | No |
|  |  | *Phlebotomus* | 29651 | No |
|  |  | *Psorophora* | 28087 | Yes |
|  |  | *Sabethes* | 4332 | No |
|  |  | *Trichoprosopon* | 1901 | No |
| Galindo, 1967 [10] | Virus isolation | *Culex* | 11829 | Yes |
| Galindo, 1983 [11] | Virus isolation | *Aedes* | NA | No |
|  |  | *Aedomyia* | NA | No |
|  |  | *Anopheles* | NA | No |
|  |  | *Culex* | NA | No |
|  |  | *Culicoides* | NA | No |
|  |  | *Haemagogus* | NA | Yes |
|  |  | *Lutzomyia* | NA | No |
|  |  | *Mansonia* | NA | No |
|  |  | *Wyeomyia* | NA | No |
| GenBank KY618129 | Virus isolation | *Haemagogus* | NA | Yes |
| GenBank KY618130 | Virus isolation | *Culex* | NA | Yes |
| Groot, 1961 [12] | Virus isolation | *Aedes* | 14524 | No |
|  |  | *Anopheles* | 355 | No |
|  |  | *Culex* | 2420 | No |
|  |  | *Haemagogus* | 444 | No |
|  |  | *Limatus* | 236 | No |
|  |  | *Mansonia* | 2218 | No |
|  |  | *Psorophora* | 20111 | Yes |
|  |  | *Sabethes* | 275 | No |
|  |  | *Trichoprosopon* | 659 | No |
|  |  | *Wyeomyia* | 322 | No |
| Henriques, 2008 [13] | RT-PCR | *Aedes* | 1971 | No |
|  |  | *Aedomyia* | 23 | No |
|  |  | *Anopheles* | 8997 | No |
|  |  | *Cerathopogonidae* | 735 | No |
|  |  | *Coquilletidia* | 2710 | No |
|  |  | *Culex* | 7864 | No |
|  |  | *Culiseta* | 12 | No |
|  |  | *Deinocerites* | 208 | No |
|  |  | *Haemagogus* | 226 | No |
|  |  | *Limatus* | 67 | No |
|  |  | *Mansonia* | 6469 | No |
|  |  | *Psychodidae* | 4820 | No |
|  |  | *Psorophora* | 2233 | No |
|  |  | *Sabethes* | 18 | No |
|  |  | *Shannoniana* | 27 | No |
|  |  | *Simuliidae* | 828 | No |
|  |  | *Tabanidae* | 139 | No |
|  |  | *Trichoposopon* | 103 | No |
|  |  | *Uranotaenia* | 15 | No |
|  |  | *Wyeomyia* | 54 | No |
| Hoch, 1981 [14] | Virus isolation | *Aedes* | 11 | No |
|  |  | *Culex* | 424 | No |
|  |  | *Culicoides* | 3609 | No |
|  |  | *Foricypomyia* | 88 | No |
|  |  | *Haemagogus* | 2284 | Yes |
|  |  | *Limatus* | 2192 | No |
|  |  | *Lutzomyia* | 574 | No |
|  |  | *Orthopodomyia* | 69 | No |
|  |  | *Psorophora* | 77 | No |
|  |  | *Sabethes* | 465 | No |
|  |  | *Trichoprosopon* | 244 | No |
|  |  | *Wyeomyia* | 630 | No |
| Kubiszeski, 2017 [15] | RT-PCR; Virus isolation | *Aedomyia* | 90 | No |
|  |  | *Aedes* | 7 | No |
|  |  | *Culex* | 634 | Yes |
|  |  | *Coquilletidia* | 2 | No |
|  |  | *Haemagogus* | 1 | No |
|  |  | *Ochelorotatus* | 26 | No |
|  |  | *Psorophora* | 2 | No |
|  |  | *Uranotaenia* | 16 | No |
| Maia, 2019 [16] | RT-PCR; Virus isolation | *Aedes* | 4786 | Yes |
| Martinez, 2020 [17] | RT-PCR | *Aedes* | 169 | No |
| Pauvolid-Correa, 2008 [18] |  | *Aedomyia* | 1 | No |
|  |  | *Amblyomma* | 30 | No |
|  |  | *Anocentor* | 40 | No |
|  |  | *Anopheles* | 350 | No |
|  |  | *Coquillettidia* | 35 | No |
|  |  | *Culex* | 156 | No |
|  |  | *Mansonia* | 1799 | No |
|  |  | *Ochlerotatus* | 14 | No |
|  |  | *Psorophora* | 1325 | No |
|  |  | *Sabethes* | 4 | No |
| Pinheiro, 1974 [19] | Virus isolation | *Aedes* | NA | No |
|  |  | *Anopheles* | NA | No |
|  |  | *Culex* | NA | No |
|  |  | *Psorophora* | NA | No |
|  |  | *Trichoprosopon* | NA | No |
|  |  | *Uranotaenia* | NA | No |
|  |  | *Wyeomyia* | NA | No |
| Pinheiro, 2019 [20] | RT-PCR | *Aedes* | 125 | No |
|  |  | *Aedomyia* | 5 | No |
|  |  | *Anopheles* | 1 | No |
|  |  | *Culex* | 46 | No |
|  |  | *Haemagogus* | 200 | No |
|  |  | *Limatus* | 166 | No |
|  |  | *Psorophora* | 273 | No |
|  |  | *Runchomyia* | 3 | No |
|  |  | *Sabethes* | 42 | No |
|  |  | *Wyeomyia* | 6 | No |
| Powers, 2006 [21] | Virus isolation | *Haemagogus* | NA | Yes |
|  |  | *Ixodes* | NA | Yes |
|  |  | *Mansonia* | NA | Yes |
| SanMartin, 1973 [22] | Virus isolation | *Aedes* | 309 | No |
|  |  | *Aedomyia* | 9 | No |
|  |  | *Anopheles* | 302 | No |
|  |  | *Culex* | 13590 | No |
|  |  | *Limatus* | 1 | No |
|  |  | *Mansonia* | 13119 | No |
|  |  | *Psorophora* | 83 | No |
|  |  | *Uranotaenia* | 3 | No |
|  |  | *Wyeomyia* | 21 | No |
| Scherer, 1975 [23] | Virus isolation | NA | 18500 | No |
| Serra, 2016 [24] | RT-PCR; Virus isolation | *Aedes* | 1089 | Yes |
|  |  | *Culex* | 3433 | Yes |
|  |  | *Galindomyia* | 1 | No |
|  |  | *Limatus* | 7 | No |
|  |  | *Mansonia* | 4 | No |
|  |  | *Psorophora* | 21 | No |
|  |  | *Sabethes* | 1 | No |
|  |  | *Uranotaenia* | 1 | No |
| Silva, 2017 [25] | RT-PCR | *Aedes* | 77 | No |
|  |  | *Aedomyia* | 3 | No |
|  |  | *Anopheles* | 209 | No |
|  |  | *Chagasia* | 7 | No |
|  |  | *Coquillettidia* | 125 | No |
|  |  | *Culex* | 862 | No |
|  |  | *Haemagogus* | 78 | No |
|  |  | *Jhonbelkinia* | 73 | No |
|  |  | *Limatus* | 81 | No |
|  |  | *Mansonia* | 3 | No |
|  |  | *Ochlerotatus* | 695 | No |
|  |  | *Psorophora* | 1230 | No |
|  |  | *Runchomyia* | 1 | No |
|  |  | *Sabethes* | 83 | No |
|  |  | *Trichoprosopon* | 12 | No |
|  |  | *Uranotaenia* | 25 | No |
|  |  | *Wyeomyia* | 186 | No |
| Tauro, 2019 [26] | RT-PCR; Virus isolation | *Aedes* | 26 | No |
|  |  | *Culex* | 99 | No |
| Taylor, 1967 [27] | Virus isolation | *Culex* | NA | Yes |
|  |  | *Gigantolaelaps* | NA | Yes |
|  |  | *Haemagogus* | NA | Yes |
|  |  | *Mansonia* | NA | Yes |
|  |  | *Sabethes* | NA | Yes |

^a^ Includes *Wyeomyia*, *Aedes*, *Sabethes*, and *Limatus*

References

1. Aitken TH, Spence L, Jonkers AH, Downs WG. A 10-year survey of Trinidadian arthropods for natural virus infections (1953-1963). J Med Entomol. 1969;6(2):207-15. Epub 1969/05/01. doi: 10.1093/jmedent/6.2.207. PubMed PMID: 5807863.

2. Azevedo RS, Silva EV, Carvalho VL, Rodrigues SG, Neto JPN, Monteiro HA, et al. Mayaro fever virus, Brazilian amazon. Emerg Infect Dis. 2009;15(11):1830. doi: 10.3201/eid1511.090461.

3. Batista PM, Andreotti R, Chiang JO, Ferreira MS, Vasconcelos PF. Seroepidemiological monitoring in sentinel animals and vectors as part of arbovirus surveillance in the state of Mato Grosso do Sul, Brazil. Rev Soc Bras Med Trop. 2012;45(2):168-73. Epub 2012/04/27. doi: 10.1590/s0037-86822012000200006. PubMed PMID: 22534986.

4. Carrera JP, Cucunubá ZM, Neira K, Lambert B, Pittí Y, Liscano J, et al. Endemic and Epidemic Human Alphavirus Infections in Eastern Panama: An Analysis of Population-Based Cross-Sectional Surveys. Am J Trop Med Hyg. 2020. Epub 2020/10/31. doi: 10.4269/ajtmh.20-0408. PubMed PMID: 33124532.

5. Catenacci LS. Abordagem one health para vigilância de arbovirus na Mata Atlântica do sul da Bahia, Brasil. [Ph.D. Thesis]. Ananindeua: Instituto Evandro Chagas; 2017. Available from: <https://patua.iec.gov.br/handle/iec/3073>.

6. Degallier N, Travassos da Rosa AP, Vasconcelos PFC, Hervé JP, Sa Filho GC, Travassos da Rosa JFS, et al. Modifications of arbovirus transmission in relation to construction of dams in Brazilian Amazonia Journal of the Brazilian Association for the Advancement of Science. 1992;44.

7. Esposito DL, da Fonseca BA. Complete Genome Sequence of Mayaro Virus (Togaviridae, Alphavirus) Strain BeAr 20290 from Brazil. Genome Announc. 2015;3(6). Epub 2015/12/19. doi: 10.1128/genomeA.01372-15. PubMed PMID: 26679574; PubMed Central PMCID: PMCPMC4683219.

8. da Silva Ferreira R, de Toni Aquino da Cruz LC, Souza VJ, da Silva Neves NA, de Souza VC, Filho LCF, et al. Insect-specific viruses and arboviruses in adult male culicids from Midwestern Brazil. Infect Genet Evol. 2020:104561. Epub 2020/09/23. doi: 10.1016/j.meegid.2020.104561. PubMed PMID: 32961364.

9. Galindo P, Srihongse S, De Rodaniche E, Grayson MA. An ecological survey for arboviruses in Almirante, Panama, 1959-1962. Am J Trop Med Hyg. 1966;15(3):385-400. Epub 1966/05/01. doi: 10.4269/ajtmh.1966.15.385. PubMed PMID: 4380043.

10. Galindo P, Srihongse S. Transmission of arboviruses to hamsters by the bite of naturally infected Culex (Melanoconion) mosquitoes. Am J Trop Med Hyg. 1967;16(4):525-30. Epub 1967/07/01. doi: 10.4269/ajtmh.1967.16.525. PubMed PMID: 4952151.

11. Galindo P, Adames A, Peralta P, Johnson C, Read R. Impacto de la hidroeléctrica de Bayano en la transmisión de arbovirus. Rev Med Pan. 1983;8:89-134.

12. Groot H, Morales A, Vidales H. Virus isolations from forest mosquitoes in San Vicente de Chucuri, Colombia. Am J Trop Med Hyg. 1961;10:397-402. Epub 1961/05/01. doi: 10.4269/ajtmh.1961.10.397. PubMed PMID: 13708940.

13. Henriques DA. Caracterização molecular de arbovírus isolados da fauna diptera nematocera do Estado de Rondônia (Amazônia ocidental brasileira) [Ph.D. Thesis]. São Paulo: Universidade de São Paulo; 2008. Available from: <https://teses.usp.br/teses/disponiveis/42/42132/tde-27032009-124003/pt-br.php>.

14. Hoch AL, Peterson NE, LeDuc JW, Pinheiro FP. An outbreak of Mayaro virus disease in Belterra, Brazil. III. Entomological and ecological studies. Am J Trop Med Hyg. 1981;30(3):689-98. Epub 1981/05/01. doi: 10.4269/ajtmh.1981.30.689. PubMed PMID: 6266265.

15. Kubiszeski JR. Arboviroses emergentes no município de Sinop-MT: pesquisa de vetores [Ph.D. Thesis]. Sinop: Universidade Federal de Mato Grosso; 2016. Available from: <https://teses.usp.br/teses/disponiveis/42/42132/tde-27032009-124003/pt-br.php>.

16. Maia LMS, Bezerra MCF, Costa MCS, Souza EM, Oliveira MEB, Ribeiro ALM, et al. Natural vertical infection by dengue virus serotype 4, Zika virus and Mayaro virus in Aedes (Stegomyia) aegypti and Aedes (Stegomyia) albopictus. Med Vet Entomol. 2019;33(3):437-42. Epub 2019/02/19. doi: 10.1111/mve.12369. PubMed PMID: 30776139.

17. Martinez D, Hernandez C, Munoz M, Armesto Y, Cuervo A, Ramirez JD. Identification of Aedes (Diptera: Culicidae) Species and Arboviruses Circulating in Arauca, Eastern Colombia. Front Ecol Evol. 2020;8. doi: 10.3389/fevo.2020.602190. PubMed PMID: WOS:000596835300001.

18. Pauvolid-Correa A. Estudo sobre arbovírus em populações de eqüinos e artrópodes na sub-região da Nhecolândia no Pantanal de Mato Grosso do Sul [M.Sc. Thesis]. Rio de Janeiro: Fundação Oswaldo Cruz; 2008. Available from: <https://www.arca.fiocruz.br/handle/icict/21142>.

19. Pinheiro FP, Bensabath G, Andrade AH, Lins ZC, Fraihi H, Tang AT, et al. Infectious diseases along Brazil's Trans-Amazon Highway: surveillance and research. Bull Pan Am Health Organ. 1974;8(111).

20. Pinheiro GG, Rocha MN, de Oliveira MA, Moreira LA, Andrade JD. Detection of Yellow Fever Virus in Sylvatic Mosquitoes during Disease Outbreaks of 2017-2018 in Minas Gerais State, Brazil. Insects. 2019;10(5). doi: 10.3390/insects10050136. PubMed PMID: WOS:000476846800018.

21. Powers AM, Aguilar PV, Chandler LJ, Brault AC, Meakins TA, Watts D, et al. Genetic relationships among Mayaro and Una viruses suggest distinct patterns of transmission. Am J Trop Med Hyg. 2006;75(3):461-9. Epub 2006/09/14. PubMed PMID: 16968922.

22. Sanmartín C, Mackenzie RB, Trapido H, Barreto P, Mullenax CH, Gutiérrez E, et al. Encefalitis equina venezolana en Colombia, 1967. Bol Oficina Sanit Panam. 1973;74(2):108-37. Epub 1973/02/01. PubMed PMID: 4265714.

23. Scherer WF, Madalengoitia J, Flores W, Acosta M. The first isolations of eastern encephalitis, group C, and Guama group arboviruses from the Peruvian Amazon region of western South America. Bull Pan Am Health Organ. 1975;9(1):19-26. Epub 1975/01/01. PubMed PMID: 238693.

24. Serra OP, Cardoso BF, Ribeiro AL, Santos FA, Slhessarenko RD. Mayaro virus and dengue virus 1 and 4 natural infection in culicids from Cuiaba, state of Mato Grosso, Brazil. Mem Inst Oswaldo Cruz. 2016;111(1):20-9. Epub 2016/01/20. doi: 10.1590/0074-02760150270. PubMed PMID: 26784852; PubMed Central PMCID: PMCPMC4727432.

25. Silva JWP. Aspectos ecológicos de vetores putativos do Vírus Mayaro e Vírus Oropuche em estratificação vertical e horizontal em ambientes florestais e antropizados em uma comunidade rural no Amazonas [M.Sc. Thesis]. Manaus, AM: Oswaldo Cruz Foundation, Instituto Leônidas and Maria Deane; 2017. Available from: <https://www.arca.fiocruz.br/handle/icict/23337>.

26. Tauro LB, Cardoso CW, Souza RL, Nascimento LC, Santos DRD, Campos GS, et al. A localized outbreak of Chikungunya virus in Salvador, Bahia, Brazil. Mem Inst Oswaldo Cruz. 2019;114:e180597. Epub 2019/03/08. doi: 10.1590/0074-02760180597. PubMed PMID: 30843962; PubMed Central PMCID: PMCPMC6396974.

27. Taylor RM. Catalogue of arthropod-borne viruses of the world: a collection of data on registered arthropod-borne animal viruses: US Public Health Service; 1967.
