# Supplementary material for: A systematic review and meta-analysis of the potential non-human animal reservoirs and arthropod vectors of the Mayaro virus
Source: PLoS Negl Trop Dis. 2021 Dec 13;15(12):e0010016. doi: 10.1371/journal.pntd.0010016 (PMC8699665; doi:10.1371/journal.pntd.0010016)
Supplement: S10 Table — (DOCX) [file pntd.0010016.s011.docx]

**S10 Table. Egger’s test for publication bias**

|  | **Studies (n)^a^** | **Pooled Prev.** | **95% CI** | **Egger Test p-value** |
| --- | --- | --- | --- | --- |
| **Primate order** | 13 | 13.1 | 4.3; 25.1 | **0.8945** |
| ***Cebus/Sapajus* genus** | 9 | 7.5 | 3.5; 15.3 | **0.4024** |
| ***Alouatta* genus** | 8 | 24.0 | 2.2; 81.6 | **0.6077** |
| **Pilosa order** | 7 | 0.0 | 0.0; 6.6 | **0.6759** |
| **Rodentia order** | 7 | 1.3 | 0.0; 6.5 | **0.6299** |
| **Domestic Equids** | 6 | 1.1 | 0.0; 4.5 | **0.3543** |
| **Didelphimorphia order** | 6 | 2.0 | 0.0; 7.2 | **0.1446** |
| **Carnivora order** | 5 | 0.1 | 0.0; 8.1 | **0.8822** |

^a^ Egger’s test was only conducted for meta-analyses that include five or more studies. Egger’s test should be interpreted with caution if 10 studies or less are included in the analysis [1]

References

1. Egger M, Davey Smith G, Schneider M, Minder C. Bias in meta-analysis detected by a simple, graphical test. BMJ. 1997;315(7109):629-34. Epub 1997/10/06. doi: 10.1136/bmj.315.7109.629. PubMed PMID: 9310563; PubMed Central PMCID: PMCPMC2127453.
