# Supplementary figures and images for: A systematic review and meta-analysis of the potential non-human animal reservoirs and arthropod vectors of the Mayaro virus
Source: PLoS Negl Trop Dis. 2021 Dec 13;15(12):e0010016. doi: 10.1371/journal.pntd.0010016 (PMC8699665; doi:10.1371/journal.pntd.0010016)

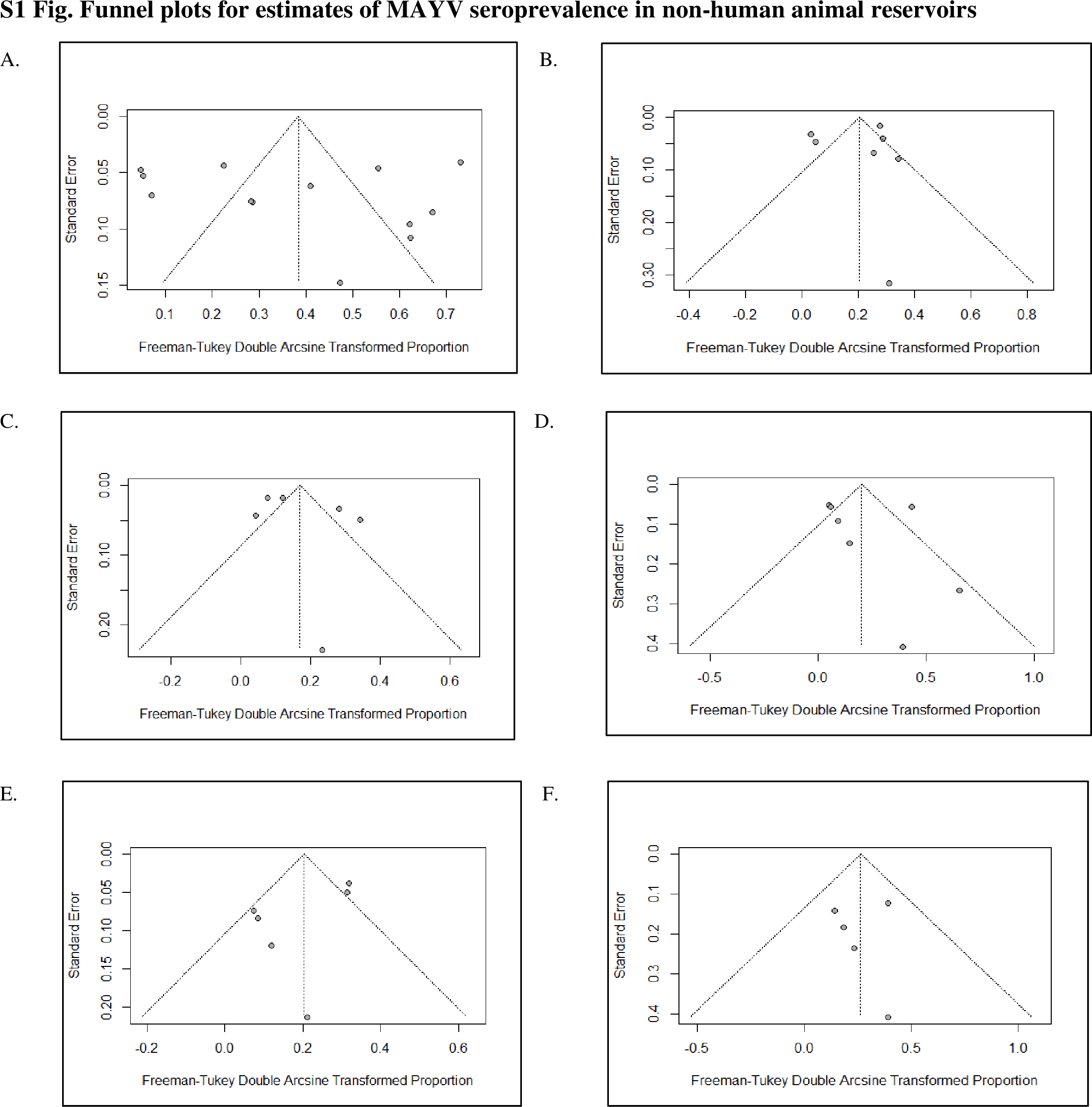

Supplement: S1 Fig — Funnel plots presented for: A) Primate order, B) Rodentia order, C) Domestic equids, D) Pilosa order, E) Didelphimorphia order, F) Carnivora order. (TIF) [file pntd.0010016.s012.tif]

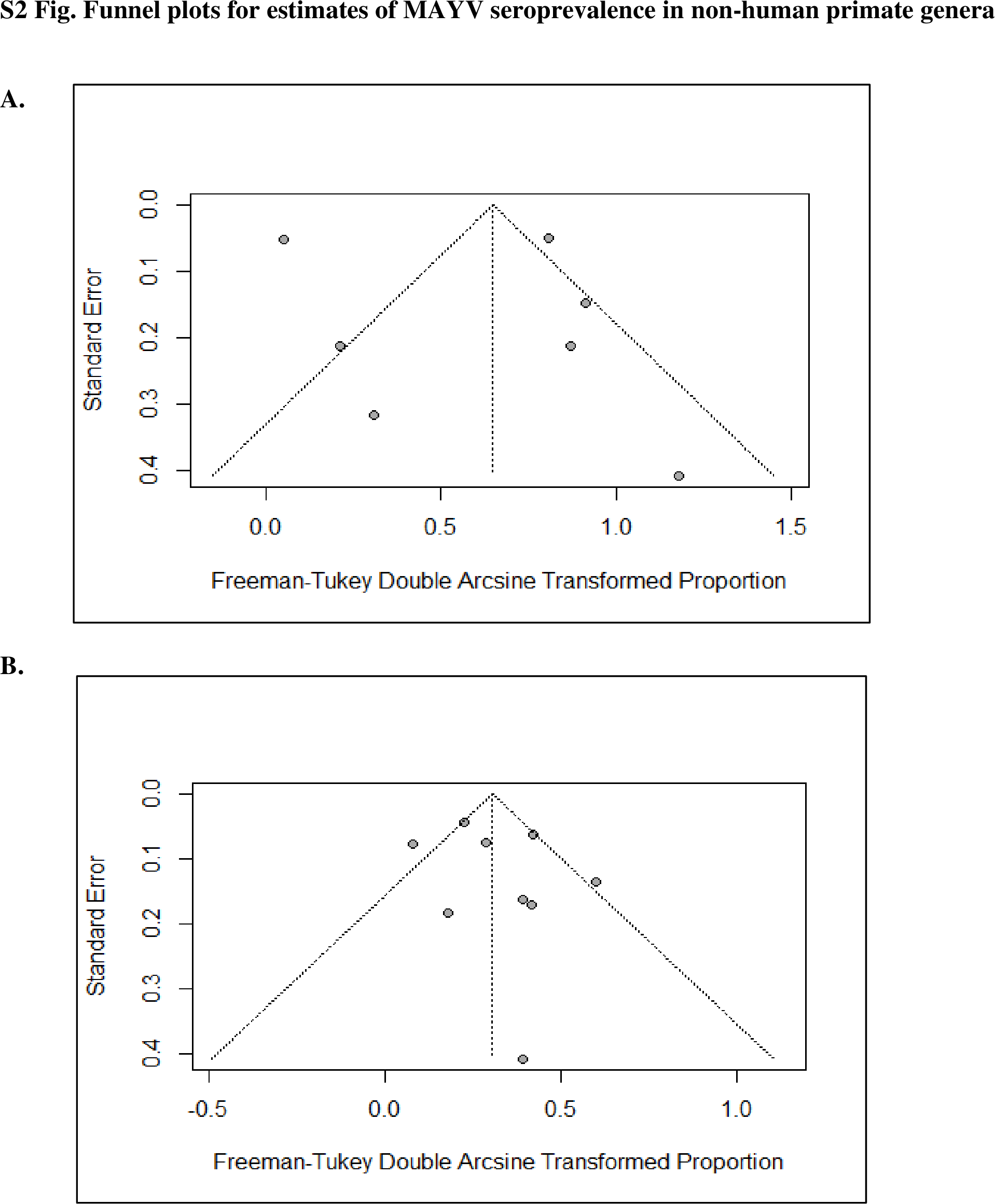

Supplement: S2 Fig — Funnel plots presented for: A) Alouatta genus and B) Cebus/Sapajus genus. (TIF) [file pntd.0010016.s013.tif]
